# Cables1 links Slit/Robo and Wnt/Frizzled signaling in commissural axon guidance

**DOI:** 10.1242/dev.201671

**Published:** 2023-10-09

**Authors:** Nikole R. Zuñiga, Alexandre Dumoulin, Giuseppe Vaccaro, Esther T. Stoeckli

**Affiliations:** ^1^ Department of Molecular Life Sciences, University of Zurich, Winterthurerstrasse 190, CH-8057 Zurich, Switzerland; ^2^ Neuroscience Center Zurich, University of Zurich, Winterthurerstrasse 190, CH-8057 Zurich, Switzerland; ^3^ University Research Priority Program (URPP) ‘Adaptive Brain Circuits in Development and Learning (AdaBD)’, University of Zurich, Winterthurerstrasse 190, CH-8057 Zurich, Switzerland

**Keywords:** Axon guidance, Floor plate, Midline crossing, β-Catenin, Wnt signaling, Spinal cord development, Neural circuit formation, Chicken

## Abstract

During neural circuit formation, axons navigate from one intermediate target to the next, until they reach their final target. At intermediate targets, axons switch from being attracted to being repelled by changing the guidance receptors on the growth cone surface. For smooth navigation of the intermediate target and the continuation of their journey, the switch in receptor expression has to be orchestrated in a precisely timed manner. As an alternative to changes in expression, receptor function could be regulated by phosphorylation of receptors or components of signaling pathways. We identified Cables1 as a linker between floor-plate exit of commissural axons, regulated by Slit/Robo signaling, and the rostral turn of post-crossing axons, regulated by Wnt/Frizzled signaling. Cables1 localizes β-catenin, phosphorylated at tyrosine 489 by Abelson kinase, to the distal axon, which in turn is necessary for the correct navigation of post-crossing commissural axons in the developing chicken spinal cord.

## Teaser

**Summary:** Commissural axons leave the floor plate and turn rostral by a smooth transition from Robo to Wnt signaling. Cables1 links these pathways by transferring Abl kinase from Robo to β-catenin.

## INTRODUCTION

During the establishment of neural circuits, axons need to connect to distant targets. On their journey, axons are directed by guidance cues provided by cells along their trajectory and from intermediate targets cutting down the long traveling distance into shorter segments. However, navigation of intermediate targets requires precise control of expression and signaling of guidance receptors (De Ramon Francàs et al., 2017; Stoeckli, 2018; Chédotal, 2019; Ducuing et al., 2019). For example, axons of the dI1 subpopulation of commissural axons cross the floor plate, the ventral midline of the spinal cord, without delay. To this end, the attractive response to the intermediate target, the floor plate, has to be turned into a repulsive response upon arrival, in order to prevent lingering of axons in the midline area, but also to prevent axon guidance errors, due to premature expression of guidance receptors sensing repulsive cues, which would prevent axons from entering the midline area.

Commissural axons are entering the floor-plate area due to the interaction between contactin-2 (also known as axonin-1) on axons and NrCAM on floor-plate cells (Stoeckli and Landmesser, 1995; Stoeckli et al., 1997). Upon contact with the floor plate, commissural growth cones start expressing Robo1 in a calsyntenin 1- and RabGDI-dependent manner (Alther et al., 2016). The temporally regulated trafficking of Robo receptors to the growth cone surface allows detection of the repulsive Slits only upon entry into the floor-plate area, preventing erroneous ipsilateral turns of axons. Expression of Robo1 on the growth cone surface thus expels axons from the floor plate (Philipp et al., 2012; Alther et al., 2016; Pignata et al., 2019).

Post-crossing axons express Hedgehog-interacting protein (Hhip) receptors, induced by Shh binding to glypican-1 (Wilson and Stoeckli, 2013), to respond to a repulsive gradient of Shh with high levels in the caudal floor-plate area (Bourikas et al., 2005). At the same time, Shh also shapes an attractive Wnt gradient along the anteroposterior axis, with higher Wnt activity levels anteriorly (Domanitskaya et al., 2010; Lyuksyutova et al., 2003). Components of both canonical and non-canonical Wnt signaling have been implicated in dI1 post-crossing commissural axon guidance along the longitudinal axis, suggesting that this strict separation of Wnt signaling into different pathways is not applicable to the role of Wnts in axon guidance (Lyuksyutova et al., 2003; Avilés and Stoeckli, 2016; van Amerongen, 2012). More recently, this has been confirmed again by a detailed study of Wnt signaling in midline crossing at the chiasm (Morenilla-Palao et al., 2020).

Despite the fact that midline crossing appears to be a rather simple, binary decision – to cross or not to cross – its regulation has been shown to be extremely complex, involving a large number of guidance cues and receptors. Because the temporal expression of these guidance receptors on growth cones has to be tightly regulated in order to ensure smooth navigation of an intermediate target, the mechanisms of receptor expression on the growth cone surface have been of great interest. In addition to the subtype-specific response of axons, as shown recently for the ipsi- versus contralaterally projecting retinal ganglion cells (Morenilla-Palao et al., 2020), signaling pathways need to be temporally regulated in the same type of axons. For example, premature expression of receptors for the morphogens presented as gradients along the longitudinal axis of the spinal cord could induce aberrant axonal decisions to turn rostrally along the ipsi- instead of the contralateral floor-plate border.

Our previous studies demonstrated that the responsiveness of only post- but not pre-crossing commissural axons to the antero-posterior Shh gradient is regulated at the transcriptional level: Hhip expression is triggered by Shh binding to glypican-1 on pre-crossing axons (Wilson and Stoeckli, 2013). Similar to the regulation of Robo1 expression, the surface expression of Fzd3, the Wnt receptors on post-crossing commissural axons, is regulated by specific vesicular trafficking (Alther et al., 2016; Onishi and Zou, 2017).

However, we reasoned that exit from the floor plate and turning into the longitudinal axis along the contralateral floor-plate border might be linked by additional mechanisms. A good candidate for such a linker between Slit/Robo signaling and Wnt signaling was Cables1 (Zukerberg et al., 2000; Rhee et al., 2007). In retinal cells, Cables1 was shown to interact with Abl kinase bound to Robo1 triggered by Slit binding, followed by Cables1-mediated transfer of Abl to β-catenin. This interaction induced dissociation of β-catenin from N-cadherin and allowed for phosphorylation of β-catenin at tyrosine residue 489 by Abl kinase (Rhee et al., 2007).

Here, we show that in the developing spinal cord Cables1 is required for midline crossing of commissural axons by linking Slit/Robo signaling to Wnt signaling involving phosphorylation of β-catenin at tyrosine 489 by Abl kinase.

## RESULTS

### Cables1 is upregulated in dI1 neurons during axonal midline crossing

**Fig. 1.**
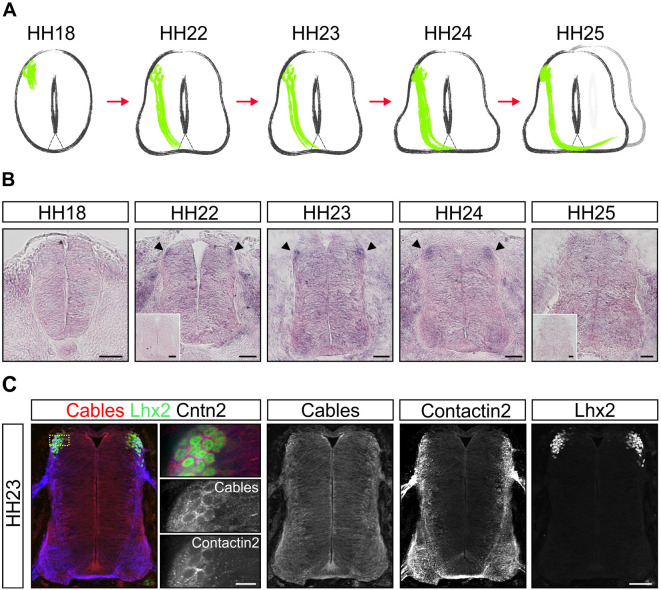
**Cables1 is upregulated in dI1 commissural neurons during axonal midline crossing.** (A) Expression of *Cables1* mRNA is shown in relationship to the temporal development of the dI1 subpopulation of commissural neurons. At HH18, dI1 commissural neurons start to extend their axons in the dorsal spinal cord. They reach and enter the floor plate at HH22. At HH24, axons exit the floor plate and turn rostral along the contralateral side of the floor plate. (B) *Cables1* mRNA is expressed at low levels throughout the developing neural tube. Higher levels are found in dI1 commissural neurons between HH22 and HH24 (arrowheads), the time window of midline crossing and axonal turning into the longitudinal axis. Expression levels in dI1 neurons decrease after midline navigation at HH25. Insert shows hybridization with the sense probe. (C) Immunostaining with an anti-Cables antibody confirms its ubiquitous expression in the developing spinal cord at HH23, when dI1 commissural axons cross the ventral midline. Cables is also found in axons crossing the floor plate, labeled with an anti-axonin-1 (contactin-2) antibody. Co-staining with an anti-Lhx2 antibody demonstrates expression of Cables1 protein in dI1 neurons. Scale bars: 50 µm (B,C); 10 µm (C, higher magnification images).

To study the role of Cables1 in commissural axon guidance we first examined the expression pattern of *Cables1*. *Cables1* mRNA was ubiquitously detected in the embryonic chicken spinal cord at different stages during commissural axon navigation (Fig. 1). The *Cables1* gene produces different proteins due to alternative splicing both in human and chicken (Zhang et al., 2005). The probe we used for *in situ* hybridization recognizes all isoforms. Using quantitative real-time PCR (qRT-PCR) to distinguish the different isoforms of Cables1 indicated that isoform X1 is the predominant splice variant in the developing spinal cord (Fig. S1A). The dI1 subpopulation of commissural interneurons showed a peak of *Cables1* expression between Hamburger–Hamilton stages (HH) 22 and HH24, which correspond to crucial points in axon navigation, entry and exit of the floor plate, the intermediate target (Fig. 1A,B; Movie 1). At HH25, *Cables1* expression in dI1 commissural neurons was reduced to almost the same level that was found throughout the spinal cord (Fig. 1B). The ubiquitous expression of Cables1 in the developing spinal cord was confirmed by immunostaining. At HH23, Cables1 protein was detected throughout the spinal cord, including the dI1 population of commissural neurons, as confirmed by Lhx2 staining (Fig. 1C). Importantly, Cables1 was also found in axons crossing the midline, stained with contactin-2. Because the antibody was raised against a domain of Cables1 that is 90% identical to the corresponding region of Cables2, and therefore does not distinguish between the two proteins, we also studied the expression of *Cables2.* Overall, Cables1 and Cables2 share 55% identity at the protein level. If at all, *Cables2* mRNA was expressed at low levels throughout the neural tube at all stages studied (Fig. S1A,B).

### Cables1 is required for axons to exit the floor plate and to turn into the longitudinal axis

The transient upregulation of Cables1 in dI1 neurons during axonal midline crossing suggested a role in commissural axon guidance. In order to test for such a role, we performed *in ovo* RNAi at HH17-18 using long dsRNA to downregulate Cables1. We evaluated the efficiency of downregulation by qRT-PCR and observed a 50% reduction for the transcript levels of *Cables1* isoform X1 24 h after electroporation (embryos sacrificed at HH23; Fig. S2A). Only 55% of the protein was left when protein levels from embryos sacrificed at HH25 were analyzed in lysates by western blotting (Fig. S2B,C). As we successfully electroporated ∼50% of the cells in the targeted area with the parameters used in this study, silencing *Cables1* by *in ovo* RNAi almost completely removed protein and transcripts in the electroporated cells.

**Fig. 2.**
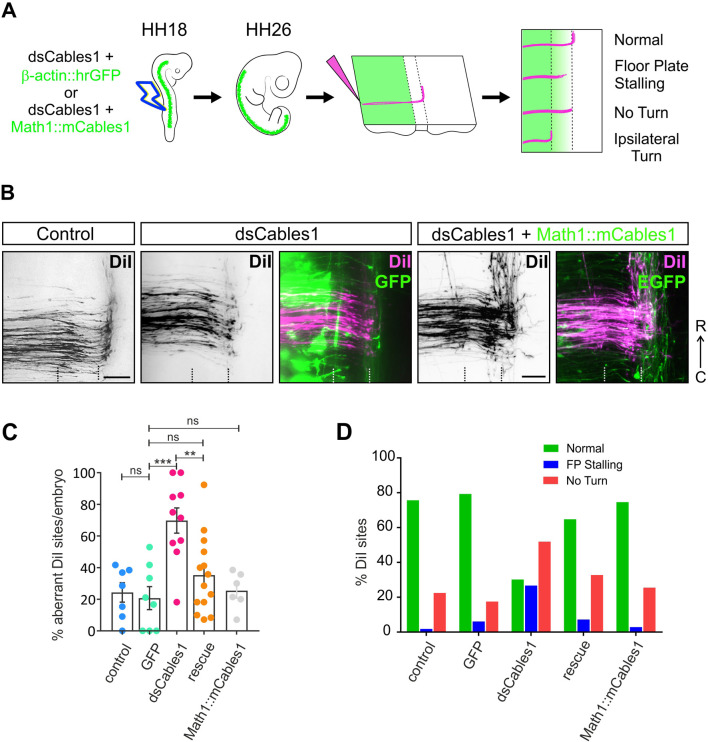
**Cables1 is required for commissural axon navigation at the floor plate.** (A) Schematic of the loss-of-function experiments using *in ovo* RNAi at HH18. Embryos were injected with dsRNA derived from *Cables1* and a plasmid encoding GFP to visualize the electroporated area of the neural tube. The trajectory of dI1 commissural axons was traced by DiI injected into open-book preparations of embryos dissected at HH26 (see Materials and Methods). For rescue experiments, the embryos were co-injected with *dsCables1* and a plasmid encoding mouse Cables1 (mCables1), which was not targeted by the dsRNA derived from chicken *Cables1*, under the control of the *Math1* enhancer for specific expression in dI1 neurons. (B) In control embryos [both untreated controls (not shown) and GFP-expressing control embryos], dI1 commissural axons cross the floor plate (indicated by dashed lines) and turn rostral along the contralateral floor-plate border. After silencing *Cables1*, most axons failed to cross the floor plate and did not turn into the longitudinal axis. The effect of Cables1 downregulation on axonal navigation was specific, because the co-injection and electroporation of *dsCables1* together with the plasmid encoding mouse Cables1 prevented the axon guidance defects seen in the absence of Cables1. (C) Quantification of the DiI injection sites with aberrant axon navigation as detailed in the Materials and Methods. The percentage of DiI injection sites with aberrant axon pathfinding did not differ when GFP-expressing control embryos (20.7±7.2%; *N*=8 embryos; *n*=93 DiI injection sites) were compared with untreated controls (24.3±6.1%; *N*=7; *n*=92). In contrast, silencing *Cables1* induced aberrant axon navigation at 69.8±8.0% of the DiI injection sites (*N*=10; *n*=103). ***P=0.0002. When mouse Cables1 was replacing chicken Cables1, only 35.2±6.5% of the DiI injection sites showed aberrant axonal navigation (*N*=14; *n*=160). This value was not significantly different (ns) from the control groups, but was significantly different from the *dsCables1* group (**P=0.0035). Data are mean±s.e.m. One-way ANOVA with Tukey's multiple comparisons test. The numbers of embryos (*N*) and injection sites (*n*) are given in parenthesis. (D) Axons in embryos electroporated with *dsCables1* mainly failed to turn along the contralateral floor-plate border, although also stalling in the floor plate was much more common than in control embryos. Scale bars: 50 μm.

For the analysis of Cables1 function, we analyzed the trajectory of dI1 commissural axons traced by injections of DiI in open-book preparations of spinal cords dissected at HH25-26 (Fig. 2A). Axons in untreated and in GFP-expressing control embryos crossed the floor plate and turned rostrally into the longitudinal axis along the contralateral floor-plate border (Fig. 2B). Only 24.3±6.1% and 20.7±7.2% (mean±s.e.m.), respectively, of the injection sites showed axons with aberrant navigation. In contrast, when Cables1 was downregulated, axons failed to turn rostrally and stalled in the floor plate or at the exit site at 69.8±8.0% of the DiI injection sites (Fig. 2B-D). These defects were due to the lack of Cables1, because we could rescue axon guidance by co-expressing mouse *Cables1* cDNA specifically in dI1 neurons using the *Math1* (*Atoh1*) enhancer (Fig. 2C,D). Under these conditions, the percentage of DiI injection sites with aberrant axonal trajectories was strongly reduced (35.25±6.5%). Injection and electroporation of the Math1::mCables1 construct alone (in control embryos) did not have an effect on axon guidance, as aberrant trajectories were seen at only 25.5±4.8% of the DiI injection sites. This is not different from non-treated or GFP-expressing control embryos.

Because we could not fully exclude the possibility that very low levels of *Cables2* were expressed throughout the developing spinal cord, including the dI1 neurons, we also silenced *Cables2*. In contrast to our findings for *Cables1*, silencing *Cables2* did not produce any aberrant phenotypes, suggesting that Cables1 function in commissural axons is specific and cannot be compensated by Cables2 (Fig. S3).

To demonstrate that the phenotype seen after perturbation of Cables1 expression was caused by the lack of axonal expulsion from the floor plate and was not due to a decrease in axonal growth speed, we also analyzed axon guidance phenotypes at HH29-30 (Fig. S4A-C). At this stage, axons were still stuck in the floor plate or failed to turn into the longitudinal axis after downregulation of Cables1, indicating that the aberrant phenotype was not simply a delay in normal axon growth. We also ruled out an indirect effect on axon guidance by aberrant neuronal differentiation (Fig. S4D).

**Fig. 3.**
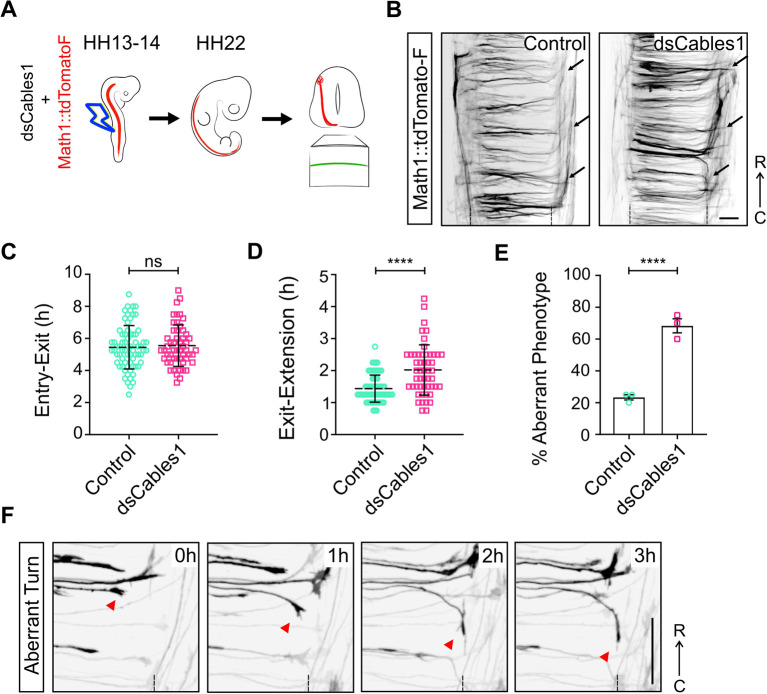
**Live imaging of dI1 commissural axons confirms turning errors of post-crossing commissural axons after silencing Cables1.** (A) Schematic depicting the design of the live imaging experiment to visualize dI1 axons crossing the ventral midline of the spinal cord in real time. First, spinal cords were unilaterally electroporated *in ovo* at HH13-14 with the dI1-specific reporter plasmid Math1::tdTomatoF with or without *dsCables1*. One day later, intact spinal cords were dissected at HH22, cultured with the ventral midline down and imaged for 24 h (1 stack every 15 min) with an inverted spinning disk microscope. (B) Examples of a temporal projection from a 24-h time-lapse recording of a control and a *dsCables1*-treated spinal cord, showing disorganization of the post-crossing segment after *Cables1* silencing compared with control (black arrows). (C) Quantification of average time of floor-plate crossing by dI1 axons did not show any significant difference in absence of Cables1 compared with control. *N* (embryos)=3 for each condition; *n* (axons)=70 (control) and 53 (*dsCables1*). (D) However, the time dI1 axons took at the floor-plate exit site to start extending along the longitudinal axis was significantly longer in the absence of Cables1 compared with control. *N*=3 for each condition; *n*=69 (control) and 51 (*dsCables1*). (E) Quantification of navigation behavior at the single axon level visualized by live imaging revealed an increase of aberrant phenotypes at the floor-plate exit site, when Cables1 was downregulated. *N*=3 for each condition; *n*=60 for each condition. (F) Example of a dI1 axon turning caudally instead of rostrally at the floor-plate exit site (red arrowhead) after downregulation of Cables1. Dashed lines represent the floor-plate boundaries (B) or floor-plate exit site (F). ****P<0.0001, ns, *P*≥0.05 (two-tailed unpaired *t*-test). Data are mean±s.e.m. R, rostral; C, caudal. Scale bars: 50 µm.

To provide additional evidence for a role of Cables1 in commissural axon guidance, we used our recently developed *ex vivo* assay to follow commissural axon navigation at the floor plate by live imaging (Dumoulin et al., 2021; Fig. 3). To this end, midline crossing by dI1 axons, visualized by Math1::tdTomato-F, was imaged in intact spinal cords embedded in an agarose gel (Fig. 3A,B). We did not see any difference in the behavior of pre-crossing axons. Also, the time it took axons to grow from entry to exit point of the floor plate was not different when we compared axons from control-treated and experimental embryos electroporated with *dsCables1* (Fig. 3B,C). However, in line with the analysis of axonal behavior in open-book preparations of spinal cords from embryos electroporated with *dsCables1* (Fig. 2B), axons were impaired at the exit site, where we observed failures to turn and aberrant turns into the longitudinal axis (Fig. 3B,D-F; Movie 2). Taken together, our results suggest that Cables1 is not required for growth of pre-crossing axons, but that it is important for axons to leave the floor plate and turn into the longitudinal axis.

### Cables1 is not required in pre-crossing commissural axons

**Fig. 4.**
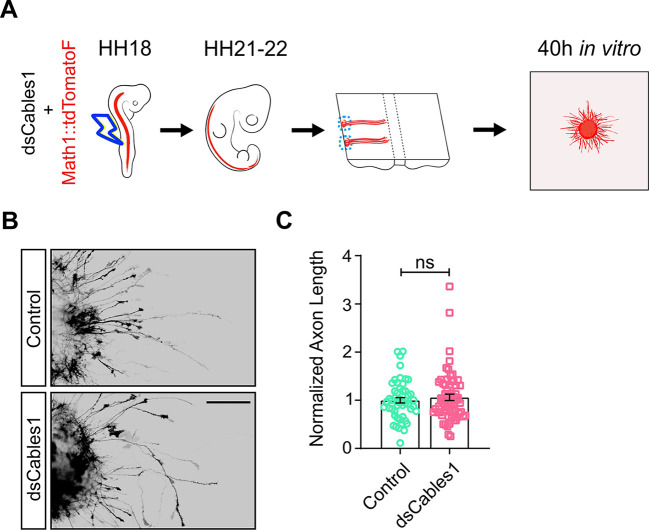
**Cables1 is not required in pre-crossing axons.** (A) We excluded an effect of Cables1 on pre-crossing axons *in vitro*. HH18 embryos were injected and electroporated with Math1::td-Tomato-F (farnesylated td-Tomato under the control of the *Math1* enhancer for specific expression in dI1 neurons) alone or together with dsRNA derived from *Cables1* (*dsCables1*). Explants of dorsal commissural neurons (blue dashed circles) were prepared from open-book preparations of spinal cords dissected from HH21/22 embryos and cultured for 40 h. (B) Axon outgrowth was visualized by staining for RFP. (C) The average lengths of commissural axons were normalized to the lengths of control explants for each experiment. A total of 52 control explants (from 13 different embryos) and 64 explants taken from embryos electroporated with *dsCables1* (*n*=16 embryos) from three independent experiments were quantified. The average length of neurites from control explants was 173 µm. See Materials and Methods for details. We did not find any significant difference (ns; *P*=0.4968) in neurite length (two-tailed unpaired *t*-test). Data are mean±s.e.m. Scale bar: 200 µm.

The absence of an effect of Cables1 on pre-crossing axons was confirmed *in vitro* (Fig. 4). We specifically labelled dI1 commissural neurons by electroporation of embryos with a plasmid encoding farnesylated td-Tomato under the control of the *Math1* enhancer (Fig. 4A). When we cultured explants of spinal cords dissected at HH21-22, we found no difference in outgrowth of td-Tomato-positive axons between experimental and control explants (Fig. 4B,C).

### Cables is required for the responsiveness of post-crossing commissural axons to Slit and Wnt5a

**Fig. 5.**
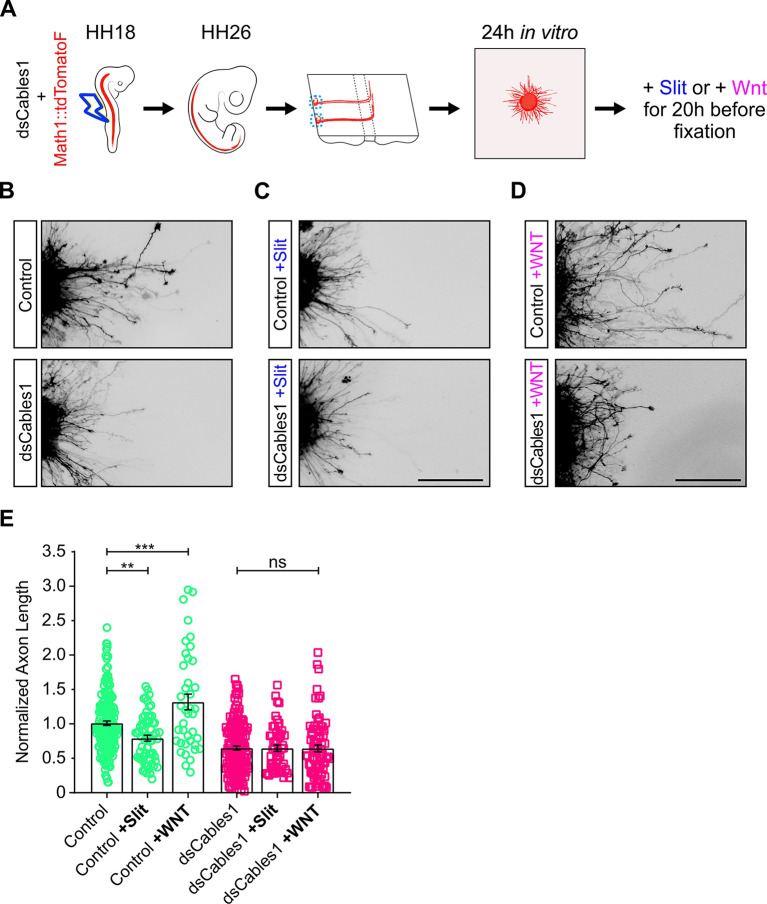
**Loss of Cables1 prevents responsiveness to Slit and Wnt5a.** (A-E) Schematic of the experimental set-up (A). HH18 embryos were electroporated with Math1::td-Tomato alone or in combination with dsRNA derived from *Cables1* (*dsCables1*). Explants of dI1 commissural neurons (blue dashed circles) were prepared from open-book preparations of spinal cords dissected from HH26 embryos and cultured for 24 h before either medium alone (control; B), Slit2 (200 ng/ml; C) or Wnt5a (200 ng/ml; D) were added to the cultures for another 20 h. Explants were fixed and stained to visualize RFP. For each experiment, neurite lengths were measured and normalized to the length of control explants (E). Data from three independent experiments are pooled. For control explants, the presence of Slit2 reduced the average axon length by 21% (*n*=16; C upper panel compared with B, upper panel). In contrast, the presence of Wnt5a increased average axon length by 32% (*n*=10; D upper panel). Axons from explants with neurons electroporated with *dsCables1* did not respond to either Slit2 (*n*=13; C lower panel) or Wnt5a (*n*=20; D lower panel). ns, not significant. **P=0.0065; ***P=0.0007 (one-way ANOVA with Tukey's multiple comparisons test). Data are mean±s.e.m. Scale bars: 200 µm.

Because dI1 commissural axons were found to stall in the floor plate *in vivo* (Fig. 2) and to fail to turn at the floor-plate exit site *in vivo* (Fig. 2) and *ex vivo* (Fig. 3), we analyzed their responsiveness to Slit and Wnt5a *in vitro*. When explants were taken from spinal cords dissected at HH26 (Fig. 5A), axon lengths were significantly shorter after addition of Slit2 to the medium (compare upper row images of Fig. 5B-D; quantified in E). As expected, based on previous results (Avilés and Stoeckli, 2016), addition of Wnt5a enhanced axon length by more than 30%. In contrast, no difference in axon lengths were found for explants containing neurons electroporated with *dsCables1* (lower rows in Fig. 5B-D, quantified in E). The average length did not change in the presence of Slit2 or in the presence of Wnt5a, indicating that Cables1 was required for the responsiveness of post-crossing commissural axons to both Slit and Wnt5a. The average length of axons extending from neurons lacking Cables1 was shorter than the length of control axons.

### Cables1 links Slit/Robo1 and Wnt/Fzd signaling *in vivo*

**Fig. 6.**
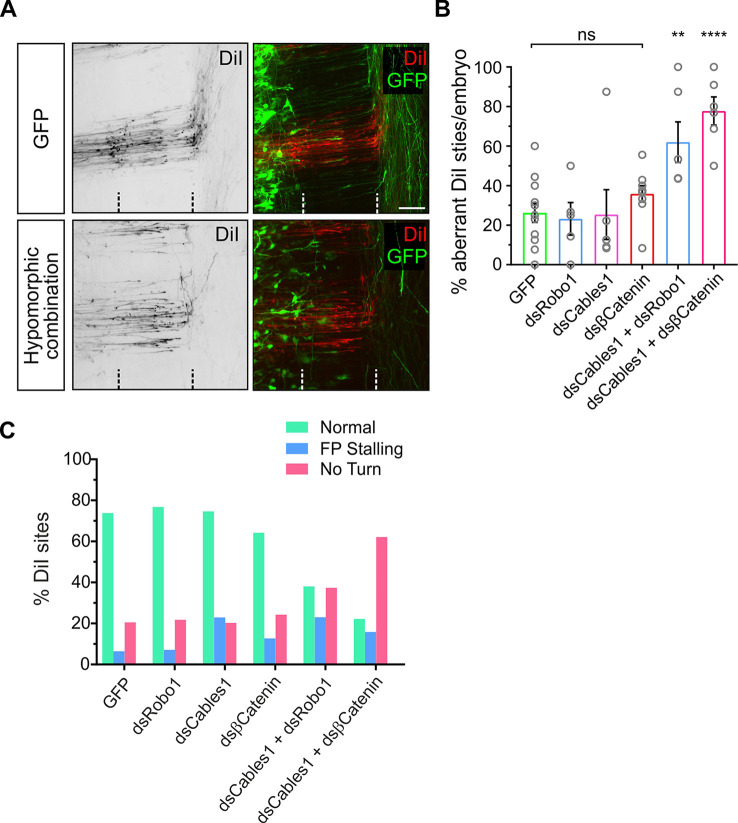
**Cables1 cooperates with Slit/Robo and Wnt/Fzd signaling in axon guidance at the midline.** (A) Open-book preparations of control (GFP-expressing) embryos and embryos electroporated with low concentrations of dsRNA derived from *Cables1*, *Robo1* or *Ctnnb1* alone or in combination were analyzed for axon guidance at the floor plate. In control embryos, axons were crossing the midline and turning rostral upon floor-plate exit. In contrast, axons failed to turn correctly or did not reach the floor-plate exit site in embryos treated with combinations of any two low concentrations of *dsCables1*, *dsRobo1* or *dsCtnnb1*. (B) Compared with GFP-expressing control embryos, where an average of 26.2±4.8% of DiI injection sites with aberrant axonal trajectories were found [*n* (injection sites)=125, *N* (embryos)=12], injection of only 75 ng/µl of dsRNA did not induce any aberrant axon guidance: *dsRobo1* (23.2±8.2%; *n*=63, *N*=5 embryos), *dsCables1* (25.4±12.5%; *n*=56, *N*=6), *dsCtnnb1* (35.8±4.1%; *n*=85, *N*=9). However, aberrant axonal navigation was found when combinations of dsRNA were used: *dsCables1*+*dsRobo1* (62.0±10.2%; *n*=85, *N*=6); *dsCables1*+*dsCtnnb1* (77.8±7.7%; *n*=70, *N*=6). ns, not significant. **P<0.01, ****P=0.0001 (one-way ANOVA with Dunnett's multiple comparisons test). Data are mean±s.e.m. (C) Injection and electroporation of only 75 ng/μl *dsRobo1*, *dsCables1* or *dsCtnnb1* alone did not significantly change the behavior of axons at the floor plate. However, the combination of *dsCables1* and *dsRobo1* resulted mostly in failure of axons to turn into the longitudinal axis at the floor-plate exit site. Note, the incidence of stalling is relatively low due to the fact that we electroporated embryos only at HH17-18. At this stage, some Robo1 is already produced and loaded into vesicles and therefore escapes functional perturbation (see Alther et al., 2016). The combination of *dsCables1* and *dsCtnnb1* resulted in a very strong effect on post-crossing axon response to Wnt signaling, as axonal turning was strongly perturbed. Scale bar: 50 µm.

Our *in vitro* results were in agreement with the hypothesis that Cables1 is required for the responsiveness to Slit and the expulsion of axons from the floor plate, but also for Wnt-dependent turning of post-crossing commissural axons along the contralateral floor-plate border. To test this idea *in vivo*, we analyzed the functional interaction of Cables1 with Robo1 and β-catenin. To this end, we used combinations of low doses of dsRNA targeting *Robo1*, *Cables1* and *Ctnnb1* that were not sufficient to induce aberrant phenotypes on their own (Fig. 6). We reasoned that if these components interacted together in the same pathway, then combinatorial partial knockdown of these genes would result in aberrant axon navigation (Fig. 6A,B). We thus lowered the concentration of the dsRNA used for electroporation that effectively interfered with axon guidance (Fig. S5) to levels that were no longer inducing significant changes in axonal behavior on their own (Fig. 6B). However, when we combined low concentrations of dsRNA targeting *Cables1* and *Robo1*, or *Cables1* and *Ctnnb1*, we found significant effects on axon guidance, indicating that these molecules act in the same pathway (Fig. 6B). As expected, the combination of *dsCables1* with *dsRobo1* had a stronger effect on midline crossing, whereas electroporation of *dsCables1* together with *dsCtnnb1* resulted in a marked increase in DiI injection sites with axons failing to turn into the longitudinal axis at the floor-plate exit site (Fig. 6C). The effect of combined downregulation of Robo1 and Cables1 would most likely be higher if the injection and electroporation of the dsRNAs were carried out at E2, as Robo1 surface expression appears to be regulated by trafficking (Alther et al., 2016). However, we wanted to use the same protocol for all groups. Taken together, our *in vivo* results obtained after combined knockdown with low concentrations of target genes confirmed a link between Slit/Robo and Wnt/Fzd signaling mediated by Cables1.

### β-Catenin is preferentially phosphorylated in the post-crossing segment of commissural axons

To confirm the role of Cables1 as a linker between Slit/Robo and Wnt/Fzd signaling and to get detailed mechanistic insight, we analyzed the distribution of phosphorylated β-catenin between pre- and post-crossing segments of commissural axons (Figs S6 and S7). Abl kinase phosphorylates β-catenin at tyrosine residue 489 (Rhee et al., 2007). Staining with a pY489-specific antibody revealed an accumulation of β-catenin pY489 in the distal, post-crossing axonal segment both *in vivo* (Fig. S6B,E) and *in vitro* (Fig. S6C,F).

**Fig. 7.**
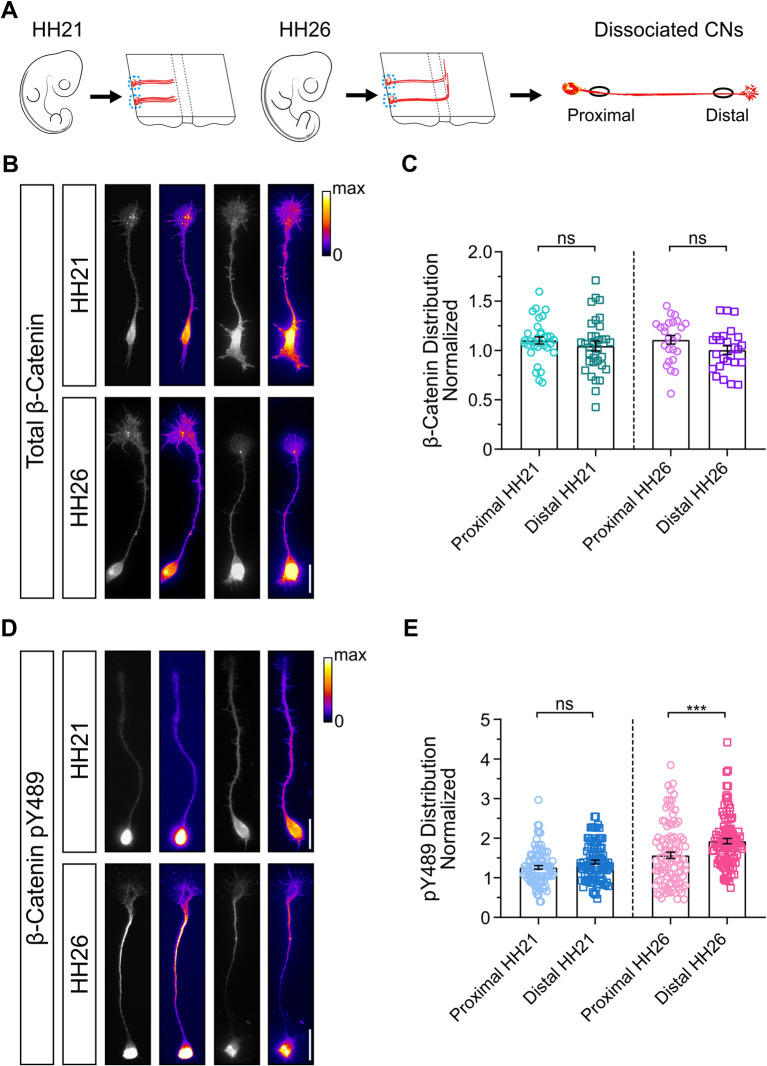
**β-Catenin pY489 accumulates in the distal part of post-crossing commissural axons.** (A-E) Schematic of the experimental design (A). Dissociated commissural neurons were prepared from the dorsal-most parts of open-book preparations of HH21 and HH26 embryos (dashed blue circles), grown for 40-48 h before staining with antibodies recognizing β-catenin or specifically β-catenin pY489. Fluorescence intensity in the proximal and distal parts of the axons (black circles) were measured and normalized to the average intensity of the entire axon. Total levels of β-catenin measured at HH21 and HH26 did not differ between the proximal and the distal axon (B,C; HH21, *n*=33 neurons; HH26, *n*=25 neurons). Similarly, comparing levels of β-catenin pY489 between proximal and distal axons of axons collected from HH21 embryos did not differ (D,E). In contrast, levels of β-catenin pY489 were significantly higher in distal compared with proximal axons of neurons collected from HH26 embryos (D,E; HH21, *n*=95 neurons; HH26, *n*=99 neurons). Results were obtained from three independent experiments. For each condition, two examples are shown in B and C demonstrating that the distribution between proximal and distal axons was consistent even when staining intensities varied between experiments. ns, not significant. ***P=0.0009 (two-tailed paired *t*-test). Data are mean±s.e.m. Scale bars: 20 μm.

Based on our results demonstrating that Cables1 had an effect on post- but not pre-crossing axons, we compared the localization of total β-catenin and β-catenin pY489 in neurons dissected from HH21 and HH26 embryos (Fig. 7A,B,D; Fig. S7). We found no difference in levels of total β-catenin between proximal and distal segments of pre-crossing or post-crossing dI1 axons (Fig. 7B,C). However, β-catenin pY489 levels were higher in distal segments of post-crossing axons (Fig. 7D,E). No such difference between proximal and distal axonal segments was seen for pre-crossing axons (HH21). In growth cones of post-crossing axons, β-catenin pY489 was predominantly found in the transition zone to the axon and in the central domain of the growth cone (Fig. S8).

**Fig. 8.**
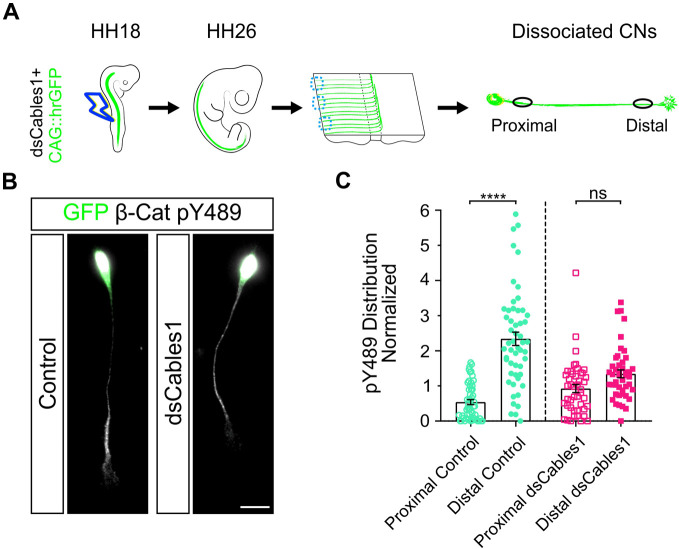
**Cables1 regulates the phosphorylation of β-catenin Y489 in post-crossing commissural axons.** (A) Schematic of the experimental design. HH18 embryos were electroporated with dsRNA derived from *Cables1* and a GFP reporter plasmid. Dissociated commissural neurons were prepared from the dorsal-most parts of open-book preparations of HH26 spinal cords (dashed blue circles), stained with an antibody specific for β-catenin pY489 and used for intensity measurements. (B) Immunostaining of commissural neurons for β-catenin pY489 isolated from control and *dsCables1*-electroporated embryos demonstrated that the accumulation of β-catenin pY489 depended on Cables1, as intensity levels no longer significantly differed when we compared distal and proximal axons (black circles) from embryos lacking Cables1. (C) Quantification of β-catenin pY489 levels in proximal compared with distal axons in control and Cables1-deficient neurons normalized to the average intensity of the entire axons. Data are mean±s.e.m. Results were obtained from three independent experiments. Control, *n*=53 neurons; *dsCables1*, *n*=45 neurons. ns, not significant. ****P<0.0001 (one-way ANOVA, separate *t*-test for control and dsCables group). The results presented here are not directly comparable with those shown in Fig. 7E, because the cultures were not stained with the same batch of anti-β-catenin pY489 antibodies. Therefore, the difference in proximal compared with distal control levels are not the same as those shown in Fig. 7E. Scale bar: 20 μm.

Next, we looked at the distribution of β-catenin pY489 in neurons lacking Cables1 (Fig. 8). The observed accumulation of β-catenin pY489 in the distal axon disappeared in the absence of Cables1, indicating that Cables1 was responsible for the accumulation of β-catenin pY489 in the distal post-crossing axons.

### Phosphorylation of β-catenin at position Y489 is required for post-crossing axon growth

**Fig. 9.**
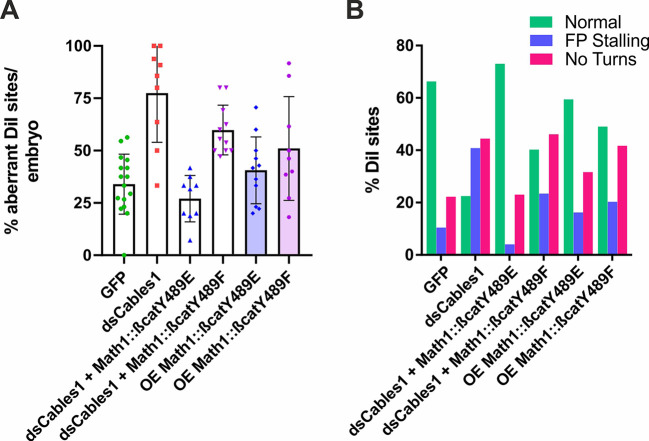
**β-CateninY489E, a constantly active mutant of β-cateninY489, rescues aberrant turning behavior of post-crossing commissural axons.** (A) The failure to turn rostral along the contralateral floor-plate border after downregulation of Cables1, seen at 77.5±7.8% of the injection sites (*N*=9 embryos; *n*=101 injection sites) compared with GFP-expressing control embryos with aberrant phenotypes at 34.0±3.6% (*N*=16 embryos, *n*=192) of the injection sites, was reduced to control levels when *dsCables1* was electroporated together with a plasmid expressing β-cateninY489E, a constantly active mutant of β-cateninY489 (27.0±3.7%; *N*=9; *n*=126). In contrast, phenotypes induced by downregulation of Cables1 could not be rescued with concomitant expression of β-cateninY489F, a mutant version of β-cateninY489 that cannot be phosphorylated at tyrosine Y489 (59.8±3.6%, *N*=11, *n*=123). The injection of the rescue constructs, Math1::β-cateninY489E (40.6±4.8%, *N*=11, *n*=135) and Math1::β-cateninY489F (51.0±8.3%, *N*=9, *n*=111) in the presence of endogenous β-cateninY489 slightly increased the DiI injection sites with aberrant axon guidance compared with control GFP-expressing embryos, but the changes were not significant (*P*=0.92 and *P*=0.18, respectively). The values were, however, significantly different from those seen after downregulation of Cables1 (*P*=0.0002 and *P*=0.0211, respectively). One-way ANOVA with Tukey's multiple comparisons test. Data are mean±s.e.m. (B) Qualitative analysis of the phenotypes indicated that expression of β-cateninY489 was not able to rescue the no-turning phenotype seen in the absence of Cables1. The percentage of DiI injection sites with post-crossing axons failing to turn after β-cateninY489 expression in control embryos was higher than controls most likely due to competition with endogenous β-catenin, despite the fact that the number of normal injection sites was not significantly different from GFP control embryos.

Next, we wanted to test our model that Cables1-mediated localization of phosphorylated β-catenin in the distal axon/growth cone was required for axon guidance *in vivo.* To this end, we generated two different β-catenin mutants: β-cateninY489E, a phosphomimetic (constantly active) mutant, and β-cateninY489F, a mutant that cannot be phosphorylated (non-active form) (Fig. 9). Both mutant forms of β-catenin were specifically expressed in dI1 neurons with the help of the *Math1* enhancer (Fig. S9). Aberrant axonal turning into the longitudinal axis in the absence of Cables1 was rescued by co-expression of β-cateninY489E, the constantly active form of β-catenin, but not with the non-active mutant of β-catenin, β-cateninY489F (Fig. 9). The overexpression of each of these mutant versions of β-catenin, Math1::β-cateninY489E and Math1::β-cateninY489F, in the presence of Cables1 did not result in axon guidance phenotypes per se. These results confirm a role of Cables1-mediated activation of β-catenin, that is phosphorylation of β-catenin at Y489, in the induction of the responsiveness to Wnt of post-crossing commissural axons.

### Robo1 is required for the phosphorylation of β-catenin at Y489

**Fig. 10.**
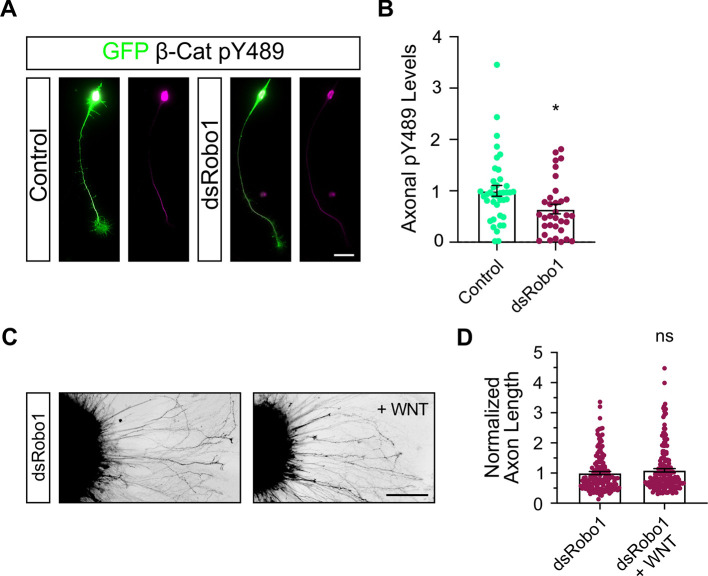
**Robo1 is required for the phosphorylation of β-catenin at Y489 and the responsiveness of axons to Wnt.** (A) To confirm a role of Robo1 in Wnt responsiveness, we stained axons dissected either from HH26 control embryos (electroporation of GFP plasmid alone) or from embryos electroporated with *dsRobo1*. (B) Levels of phosphorylated β-catenin (β-catenin pY489) were reduced in the absence of Robo1. Control, *n*=40 neurons; *dsRobo1*, *n*=33 neurons. *P=0.0145 (two-tailed unpaired *t*-test). Results were combined from three independent experiments. (C) This observation was in line with the failure of axons to respond to Wnt5a (200 ng/ml) added to explants taken from embryos electroporated with *dsRobo1*. (D) The average axon length in the absence of Robo1 did not increase after addition of Wnt5a. *dsRobo1*, *n*=45 explants; *dsRobo1*+Wnt5a, *n*=51 explants. ns, not significant. Compare with Fig. 5 for the effect of Wnt5a addition to control explants. Data are mean±s.e.m. Scale bar: 20 µm (A); 200 µm (C).

To provide additional evidence for our findings that Cables1 links Slit/Robo1 and Wnt signaling in dI1 axon navigation, we tested the necessity for Robo1 in the phosphorylation of β-catenin at Y489 (Fig. 10). Axons taken from embryos electroporated with *dsRobo1* showed lower levels of β-catenin pY489 (Fig. 10A,B). Finally, we further tested the link between Slit/Robo and Wnt signaling by exposing explants of dI1 neurons to Wnt5a (Fig. 10C). In contrast to neurons taken from control embryos (see Fig. 5D,E), neurons from embryos electroporated with *dsRobo1* did not respond to Wnt5a added to the medium (Fig. 10C,D).

**Fig. 11.**
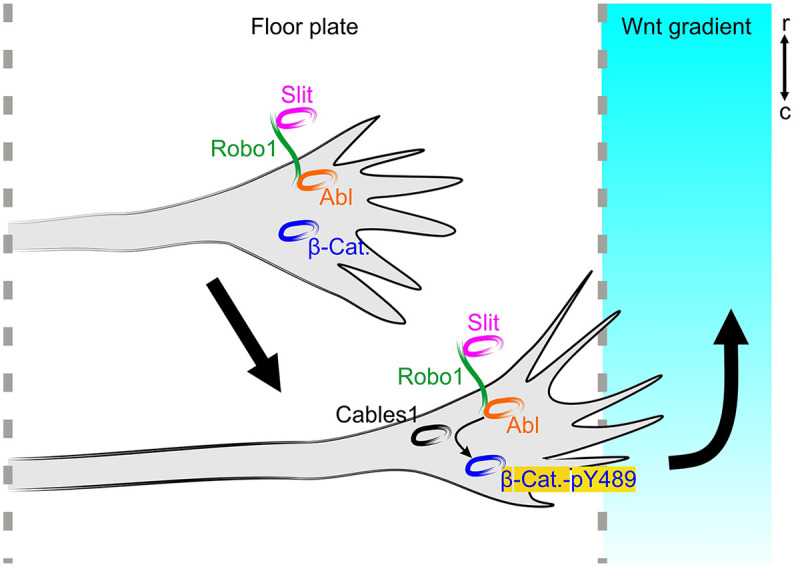
**Cables1 links Robo-mediated floor-plate exit with responsiveness to Wnt signaling by regulating Abl-mediated phosphorylation of β-catenin.** Commissural axons are expelled from the floor plate (indicated by dashed lines) by the midline-associated repellent Slit binding to Robo1 on the growth cone surface. Cables1 controls the exit of commissural axons from the floor plate by transferring Abl kinase from Robo1 to β-catenin. This results in phosphorylation of β-catenin at tyrosine 489 (β-Cat.-pY489) and its accumulation in the distal axon and the central domain of the growth cone. Accumulation of β-catenin pY489 in the distal axon and growth cone is required for the responsiveness of post-crossing commissural axons to the Wnt gradient. r, rostral; c, caudal.

Taken together, our *in vivo* and *in vitro* data support the model (Fig. 11) that Cables1 links Slit/Robo signaling during midline crossing with Wnt/Fzd signaling required for post-crossing axons to turn rostral in response to the Wnt gradient upon floor-plate exit. Cables1 is required for the localization of phosphorylated β-catenin pY489 in distal axons and growth cones, which in turn is required for correct turning of post-crossing commissural axons along the antero-posterior Wnt gradient.

## DISCUSSION

Receptor expression on the growth cone surface has to be precisely controlled for different stages of axonal pathfinding, as different receptors are involved in axonal navigation towards the intermediate target, arrival versus departure without lingering, and continuation of growth towards the next intermediate or the final target. Most studies have concentrated on the regulation of receptor expression for a particular signaling pathway. In contrast, this study describes how two known signaling pathways are connected. Using *in vivo* and *in vitro* assays, we identified a role for Cables1 as a linker between the Slit/Robo-mediated floor-plate exit of dI1 commissural axons and their Wnt/Fzd-mediated turn into the longitudinal axis. Previous studies have demonstrated that expression of Robo1, the receptor for the repulsive Slit molecules expressed by the floor plate, is regulated at the post-translational level (Alther et al., 2016; Pignata et al., 2019; Kinoshita-Kawada et al., 2019). This mechanism is in line with descriptions in flies, where Robo expression was shown to depend on trafficking as well (summarized by Gorla and Bashaw, 2020). In vertebrates, miRNA-mediated regulation of translation has been identified as an additional regulatory mechanism of Robo surface expression (Yang et al., 2018).

Responsiveness to the guidance cues regulating axon guidance along the longitudinal axis of the spinal cord is controlled by different mechanisms. The expression of Hhip, the Shh receptor on post-crossing axons, is regulated at the transcriptional level by Shh itself in a glypican-1-dependent manner (Bourikas et al., 2005; Wilson and Stoeckli, 2013). In contrast, expression of Fzd3, the Wnt receptor on post-crossing axons, is regulated by specific trafficking (Alther et al., 2016; Onishi and Zou, 2017). Robo and Fzd receptors are transported to the growth cone surface in a calsyntenin 1-dependent manner but in different vesicles (Alther et al., 2016). Timing of vesicular transport of Robo1 and its insertion into the growth cone membrane has been shown to depend on RabGDI (Philipp et al., 2012; Alther et al., 2016). The timer for Fzd3 transport and insertion is still elusive, as RabGDI is not involved in Fzd3 expression on the growth cone surface.

Here, we characterized the role of Cables1 in dI1 commissural axon guidance at the floor plate. Our studies demonstrate a novel regulatory mechanism and fine-tuning of the temporal sequence of events. Cables1 acts as a molecular linker between the Slit/Robo and the Wnt/Fzd pathway. Our results demonstrate that Cables1 is required for axon guidance (Fig. 2) rather than just axonal growth (Figs 3 and 4; Fig. S4) or neuronal differentiation (Fig. S4D), although growth and guidance cannot be separated for post-crossing axons (Figs 2 and 5). Post-crossing axons are shorter *in vitro* and fail to respond to Wnt5a in the absence of Cables1 (Fig. 5).

Our results are in line with a model that suggests an association between Abl and Robo1 in axons crossing the midline (Rhee et al., 2007, but see also Bashaw et al., 2000). Upon Slit binding, Robo1 receptors are internalized and the associated Abl molecule is detached from Robo via Cables1. Cables1 brings Abl in close proximity to β-catenin, which gets phosphorylated at tyrosine residue 489. This phosphorylation changes the interactions of β-catenin and prepares it for its role in Wnt signaling at the floor-plate exit site.

Our study is the first report on the involvement of a Robo/Cables1/β-catenin link in commissural axon guidance. So far, Robo-mediated expulsion of axons from the Slit-expressing floor plate has not been functionally linked to guidance cues for the longitudinal axis. We previously demonstrated a role of β-catenin in Wnt signaling and guidance of post-crossing commissural axons (Avilés and Stoeckli, 2016). Here, we extend these findings and demonstrate that β-catenin needs to be phosphorylated at tyrosine 489 for its role in post-crossing axon guidance (Fig. 9). This finding is intriguing in the context of a recent study about axonal navigation at the chiasm (Morenilla-Palao et al., 2020). The authors found phosphorylation of β-catenin at tyrosine 654 in ipsilaterally projecting retinal ganglion cell axons. In contrast to Abl-mediated β-catenin phosphorylation in post-crossing dI1 axons, which is on tyrosine 489, ipsilaterally projecting axons in the visual system are phosphorylated by EphB1. In both cases, crossing the chiasm and crossing the floor plate requires β-catenin, as silencing β-catenin resulted in axonal stalling at the midline (Morenilla-Palao et al., 2020; Avilés and Stoeckli, 2016; this study).

Taken together, our *in vivo* and *in vitro* results suggest a model for Cables1 function that connects the Robo1-mediated exit from the floor plate in response to Slit binding with the attractive effect of Wnts directing post-crossing axons rostrally (Fig. 11). Cables1 links Robo1-bound Abl kinase to β-catenin. The accumulation of Abl-dependent phosphorylation of β-catenin at tyrosine 489 (Y489) in the growth cone/distal axon is required for post-crossing axons to respond to Wnt5a. Our results demonstrate that Cables is required for the distal localization of phosphorylated β-catenin-pY489 (Fig. 8). In turn, co-electroporation of a dominant active form of β-catenin-pY489, β-catenin-Y489E, can rescue the lack of Cables1 (Fig. 9), indicating that β-catenin-pY489 is required for Wnt responsiveness of post-crossing axons upon floor-plate exit. In turn, phosphorylation of β-catenin at Y489 requires Robo signaling (Fig. 10). Taken together, our experiments suggest Cables1 as a linker between Robo/Slit and Wnt signaling to ensure smooth navigation of commissural axons out of the floor plate and rostral along the contralateral floor-plate border.

## MATERIALS AND METHODS

### Animals

Fertilized chicken (*Gallus gallus*) eggs were obtained from a local supplier and incubated at 39°C. All the experiments including chicken embryos were carried out in accordance with Swiss law on animal experimentation and approved by the cantonal veterinary office of Zurich.

### *In ovo* electroporation

After 2 or 3 days of incubation at 39°C, fertilized eggs were windowed for injection and electroporation, as described previously (Wilson and Stoeckli, 2011, 2012). Embryos were staged according to Hamburger and Hamilton (1992). Unilateral electroporations were performed at embryonic day (E) 3, HH17-18, using five pulses of 25 V of 50 msec duration and 1 s interpulse interval.

### Plasmids and dsRNA

For functional gene analysis, chicken embryos were injected and electroporated with long dsRNA (300 ng/µl) derived from the target gene (500 ng/µl for *dsCables1*) and a plasmid encoding β-actin-driven hrGFP (25 ng/µl). For hypomorphic experiments, combination of low doses (75 ng/µl) of each dsRNA were used (see Table S1 for details).

For the pY489 phospho-mutant versions of β-catenin, we used the Q5 Site-directed mutagenesis kit (New England Biolabs) to generate β-cateninY489F, a form of β-catenin that cannot be phosphorylated at tyrosine 489 due to the exchange of tyrosine 489 with phenylalanine, and β-cateninY489E (exchange of tyrosine 489 with glutamic acid), a phosphomimetic form of β-catenin that is dominant active. The following primers were used for the phosphomimetic substitution of Tyr (tat) by Glu (gaa): Fw, 5′-TCGCCTTCATcaaGGACTGGCCTGTTG-3′; Rv, 5′-ACGGCATTCTGGGCCATC-3′. For the phosphoinhibited substitution of Tyr (tat) by Phe (ttt): Fw, 5′-TCGCCTTCATtttGGACTGCCTG-3′; Rv, 5′-ACGGCATTCTGGGCCATC-3′. For rescue experiments, the open reading frame of mouse Cables1 was obtained from Biocat/Origene. The amplified PCR fragment was subcloned via HIFI cloning (New England Biolabs) under a *Math1* enhancer.

### Open-book preparations and DiI tracing

Spinal cords were dissected at HH25-26 (E5) as open-book preparations and fixed for 30 min in 4% paraformaldehyde (PFA) in PBS. To label dI1 commissural neurons, we injected Fast-DiI (5 mg/ml in ethanol; Thermo Fisher Scientific) into the area of the cell bodies in the dorsal spinal cord, as described previously (Wilson and Stoeckli, 2012; Perrin and Stoeckli, 2000). The trajectory of dI1 axons at each DiI injection site was analyzed by a person unaware of the experimental condition and categorized as ‘normal’, ‘floor-plate stalling’ or ‘no turns’. A ‘normal’ phenotype consists of axons entering and crossing the floor plate with an exclusively rostral turn into the longitudinal axis of the spinal cord at the floor-plate exit site. When at least 50% of the DiI-labelled axons failed to reach the contralateral floor-plate border, the DiI-injection site was counted as ‘floor-plate stalling’. When >50% of the axons reaching the exit site failed to turn rostral, the DiI injection site was classified as showing a ‘no turn’ phenotype. If less than ten axons reached the floor-plate exit site, we did not assess the turning phenotype at this site. For quantification, we calculated the ratio of DiI injection sites per embryo with either a ‘normal’, a ‘floor-plate stalling’ or a ‘no turn’ phenotype. However, for statistical analyses between different experimental groups, we combined the different aberrant phenotypes into one group, as the two phenotypes (‘floor-plate stalling’ and ‘no turns’) are not independent of each other. At a DiI injection site where all or almost all axons failed to reach the floor-plate exit site, a failure to turn cannot be assessed. Therefore, in our analysis of dI1 trajectories, the number of DiI injection sites with aberrant turning in experimental groups are likely to be underestimated. We would also like to stress that our quantification is independent of the actual number of axons labelled by the injection of DiI. It was always very easy to assess whether the majority of the axons crossed and turned, and it was always the same person analyzing the trajectories of dI1 axons.

For embryos dissected at HH29-30 (E6), ipsilateral turns or ipsilateral stalling was observed, but excluded from the quantification, as later developing populations of axons labelled by late DiI injections normally extend to the floor plate without crossing. Therefore, these normally navigating axons could not be distinguished from dI1 axons stalling at the floor-plate entry site. Images were acquired using an Olympus BX61 microscope equipped with a spinning disk unit. Data are given as mean±s.e.m. Statistical analysis was performed using Prism 8 (GraphPad).

### *In situ* hybridization and immunostaining

Embryos were sacrificed and fixed in 4% PFA in PBS at room temperature for different times depending on the stage. The tissue was cryoprotected by incubation in 25% sucrose/PBS and then embedded in Tissue-Tek O.C.T. Compound (Sakura). Specimens were frozen in isopentane on dry ice and stored at −20°C. Sections of 25 μm thickness were obtained using a cryostat (Leica, CM1850). Expressed sequence tags (ChESTs; SourceBioScience, Table S1) were used to generate *in situ* probes using a DIG RNA labeling kit (Roche). *In situ* hybridization was performed as previously described (Mauti et al., 2006). Immunostaining was performed as previously described (Perrin et al., 2001; Wilson and Stoeckli, 2011). The complete list of ChESTs and antibodies can be found in Table S1.

For immunostaining, cultures of dissociated neurons and explants were fixed in 4% PFA for 15 min at room temperature. Cells and explants were incubated in 100 mM glycine for 20 min and permeabilized with PBST (0.25% Triton X-100 in PBS) for 15 min. To reduce unspecific binding of antibodies, cells/explants were incubated in 10% fetal calf serum (FCS) in PBST at room temperature for 30 min. Primary antibodies diluted in 10% FCS/PBST were incubated at 4°C overnight. The following day, cultures were washed twice in PBST and incubated with secondary antibodies diluted in 10% FCS/PBST for 2 h at room temperature. Before mounting in Mowiol-DABCO, samples were washed three times in PBS.

### Quantitative real-time-PCR

RNA was isolated from spinal cords of embryos at HH22 and HH25 using the RNeasy mini kit (#74134, Qiagen). For the evaluation of RNAi efficiency, chicken embryos were electroporated at HH17-18 and neural tubes were dissected 24 h later under a fluorescence microscope (Olympus, SZX12). Total RNA was then reverse transcribed using SuperScript™ III First-Strand Synthesis SuperMix (#18080-400, Thermo Fisher Scientific). The primers used for the qRT-PCR reaction are listed in Table S1. qRT-PCR was performed using the Fast Sybr Green Master Mix (#4385610, Thermo Fisher Scientific) and run on a QuantStudio 3 Real Time PCR System (Applied Biosystems). mRNA expression levels were normalized to the expression level of chicken 18S ribosome (Himmels et al., 2017) and quantified using the 2^−ΔΔCt^ method. PCR amplifications were assessed from pools of spinal cords from at least three independent experiments. For quantification of *Cables1* isoform levels and *Cables2*, values were normalized to HH22 *Cables1_X1*.

### SDS-PAGE and western blotting

For the evaluation of RNAi efficiency, chicken embryos were electroporated at HH17-18 with a combination of two different dsRNAs targeting Cables1 (500 ng/µl each). Neural tubes were dissected 48 h later under a fluorescence microscope (Olympus, SZX12). Cells were lysed with RIPA buffer (150 mM NaCl, 1% Nonidet P-40, 0.5% sodium deoxycholate, 0.1% SDS, 50 mM Tris-HCl, pH 7.4) supplemented with protease inhibitors (Roche, 11836170001) and phosphatase inhibitors (5 mM NaF, 1 mM Na_3_VO_4_, 10 mM β-glycerophosphate). Protein concentrations were measured and samples were prepared for PAGE by adding 0.2 volumes of 5× Loading Buffer (650 mM Tris-Cl, pH 6.8, 5% SDS, 25% glycerol, 500 mM DTT and bromophenol blue) and incubated for 5 min at 95°C. Protein samples were separated by SDS-PAGE and transferred to a PVDF membrane. The membranes were blocked with 5% milk in TBST (0.01 M Tris-HCl, pH 7.5, 150 mM NaCl, 0.1% Tween20), followed by primary antibody incubation overnight at 4°C. On the following day, membranes were washed in TBST three times for 15 min, before incubation for 2 h at room temperature with the corresponding secondary antibodies conjugated to horseradish peroxidase. Membranes were washed in TBST before using the ECL Western Blotting Detection Reagent (GE Healthcare). The chemiluminescence signal was detected using the Amersham Imager 600 (GE Healthcare). See Table S1 for antibodies.

### Quantification of axonal outgrowth from commissural explants

For average length measurements, only td-Tomato-F-positive dI1 axons (labeled by electroporation of embryos with Math1::td-TomatoF) were used. Each explant was divided into four quadrants and the average neurite length from the explant border was measured for each quadrant using ImageJ (v 1.52i, Java 1.8.0_101 64-bit, National Institutes of Health). Axons were manually traced using a Wacom DTU-1931 tablet and pen tool. Data from at least three different independent experiments were pooled and normalized to control conditions of each independent experiment. Data are given as mean±s.e.m. Statistical analysis was performed using Prism 8 (GraphPad).

### Quantitative analysis of phospho-Y489 levels in commissural neurons

To analyze total β-catenin and β-catenin-pY489 levels in commissural neurons, we performed fluorescence intensity measurements using ImageJ. Axons were carefully delineated (excluding the growth cone) to acquire mean levels of fluorescence. For comparison of axonal distribution, we measured the mean fluorescence in a determined area in the distal and proximal axon and normalized to total axon levels. To account for background signal, we measured the mean fluorescence value by selecting an area adjacent to the axon. For quantification, at least 20 neurons per condition were measured using a Wacom DTU-1931 tablet and pen tool. Data from at least three different independent experiments are pooled and normalized to control conditions of each independent experiment. Data are given as mean±s.e.m. Statistical analysis was performed using Prism 8 (GraphPad).

### Primary neuron cultures and explant cultures

Explants of commissural neurons were obtained from dorsal spinal cords dissected from HH25-26 embryos for post-crossing and HH21-22 for pre-crossing neurons. To ensure that we dissected dI1 commissural neurons, embryos were dissected under a fluorescent stereoscope in order to visualize Math1::td-TomatoF-positive cells. Commissural explants were grown on eight-well LabTek slides (Nunc) coated with poly-Lysine (20 μg/ml; Sigma-Aldrich) and laminin (10 μg/ml). The medium for commissural neurons was as previously described (Niederkofler et al., 2010), except for pre-crossing cultures, where the medium was supplemented with recombinant Netrin (100 ng/ml, R&D Systems).

For experiments assessing Slit and Wnt responsiveness, control medium or medium containing Slit2 (200 ng/ml; R&D Systems) or Wnt5a (200 ng/ml; R&D Systems) was added to the commissural neurons after 48 h or to the explants after 24 h *in vitro*. Explants were grown for an additional 20 h before fixation and immunostaining.

For cultures of dissociated commissural neurons, neurons were obtained from dorsal spinal cords dissected from HH25-26 embryos for post-crossing and HH21 for pre-crossing cultures. Commissural neurons were grown on eight-well LabTek slides (Nunc) coated with poly-L-Lysine (20 μg/ml; Sigma-Aldrich) and laminin (10 μg/ml). The culture medium was as previously described (Niederkofler et al., 2010), except for pre-crossing cultures where the medium was supplemented with recombinant Netrin (50 ng/ml, R&D Systems). Primary neurons were plated at low density (8000-10,000 cells/well) and kept in an incubator with 5% CO_2_ at 37°C. Cultures were grown for 40-48 h before fixation and immunostaining.

### Live imaging of cultured intact spinal cords

Live imaging of intact spinal cords was performed as previously described (Dumoulin et al., 2021). Plasmids and dsRNA were injected *in ovo* into the central canal of the neural tube and electroporated unilaterally at either HH13-14 (Cables1 knockdown experiments; 700 ng/µl Math1::tdTomato-F and 30 ng/µl β-actin::EGFP-F±500 ng/µl *dsCables1*; Fig. 3) or HH17 (mRuby3-mCables 1 overexpression; 700 ng/µl Math1::EGFP-F and 1000 ng/µl mRuby3-mCables1; Movie 1) with a BTX ECM830 square-wave electroporator (five pulses at 18 or 25 V with 50 ms duration each) One day later, intact spinal cords were dissected from HH22 embryos and embedded with the ventral side down in a 100-µl drop of 0.5% low-melting agarose-culture medium mix in a 35-mm Ibidi µ-Dish with glass bottom (Ibidi, #81158). Then 200 µl of culture medium [MEM with Glutamax (Gibco)] supplemented with 4 mg/ml Albumax (Gibco), 1 mM pyruvate (Sigma-Aldrich), 100 units/ml penicillin and 100 µg/ml streptomycin (Gibco) were added on top of the agarose drop. Intact spinal cords were incubated for 30 min at 37°C, 5% CO_2_ and 95% air in a PeCon cell vivo chamber before time-lapse recordings were started. Live imaging recordings were acquired using an Olympus IX83 inverted microscope equipped with a spinning disk unit (CSU-X1 10,000 rpm, Yokogawa). Levels of CO_2_ and temperature were controlled by the cell vivo temperature controller and the CO_2_ controller units (PeCon). For the *dsCables1* experiments (Fig. 3), 30-40 planes (1.5 µm spacing) of 2×2 binned *z*-stack images were taken every 15 min for 24 h with a 20× air objective (UPLSAPO 20×/0.75, Olympus) and an Orca-Flash 4.0 camera (Hamamatsu) with the help of Olympus CellSens Dimension 2.2 software. For the mRuby3-mCables experiment (Movie 1) recordings were performed with a 40× silicone oil objective (UPLSAPO S×40/1.25, Olympus) with one stack taken every 10 min. Data acquired with 40× magnification were 3D deconvolved with a constrained iterative deconvolution of the Olympus CellSens Dimension 2.2 software (five iterations with adaptive PSF and background removal, Olympus). *Z*-stacks and maximum projections of *z*-stack movies were modified and assembled using Fiji/ImageJ (Schindelin et al., 2012). Virtual tracing of single dI1 axons crossing the floor plate (Fig. 3) was performed in Fiji using the MtrackJ plugin (Meijering et al., 2012) as previously described (Baeriswyl et al., 2021). Axon behavior was counted as aberrant when instead of turning rostrally there was either caudal turning, stalling or overshooting at the floor-plate exit site.

## Supplementary Material

10.1242/develop.201671_sup1 Supplementary information
