# Supplementary material for: Cables1 links Slit/Robo and Wnt/Frizzled signaling in commissural axon guidance
Source: Development. 2023 Oct 9;150(19):dev201671. doi: 10.1242/dev.201671 (PMC10617602; doi:10.1242/dev.201671)
Supplement: Supplementary information [file develop-150-201671-s1.pdf]

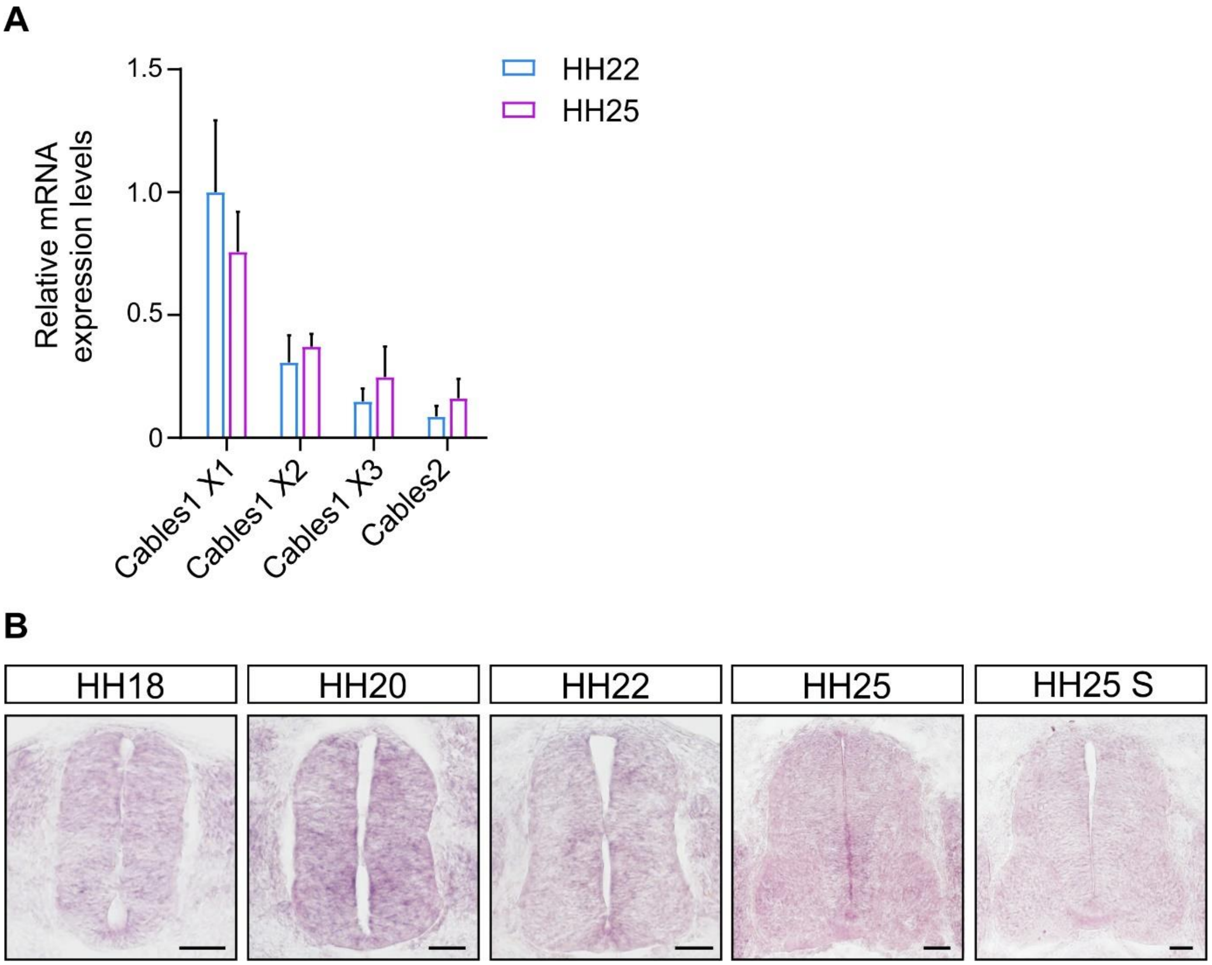

Fig. S1. In the developing spinal cord, Cables2 is expressed at very low levels, if at all

qRT-PCR analysis showed high expression levels of Cables1 isoformX1 in comparison to Cables1 isoformX2 and isoformX3, as well as Cables2 at stages HH22 and HH25. All mRNA levels were normalized to Cables1 isoformX1 at HH22 (A). If at all, Cables2 mRNA was found at very low levels throughout the developing neural tube during the time window, when dI1 commissural axons cross the floor plate and turn rostral along the contralateral floor-plate border (compare sections hybridized with anti-sense probe to the section hybridized with the sense probe (last panel labeled S)). In contrast to what we observed for Cables1 mRNA (Figure 1), Cables2 mRNA was not upregulated in dI1 commissural neurons during the time when their axons cross the midline (B). Scale bar: 50 µm.

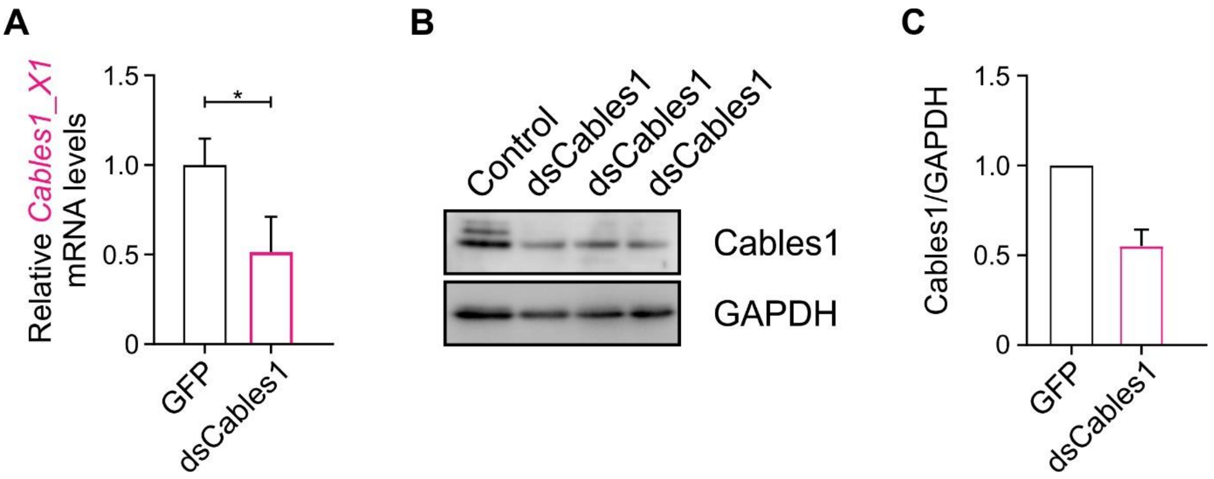

Fig. S2. Downregulation of Cables1 by in ovo RNAi effectively reduces levels of Cables1 mRNA and protein.

Effective downregulation of Cables mRNA was demonstrated with qRT-PCR for isoform X1 (A) with 4 pools of embryos sacrificed at HH23 (A). *p=0.03, paired t-test. Western blotting of proteins isolated from HH25 spinal cords confirmed effective downregulation of Cables1 in three independent pools of embryos electroporated with dsCables1 at HH17/18 (B). Levels were reduced by 50-60%. With the parameters used to silence Cables1, we successfully electroporated on average 53% (range 45-60%) of the cells in the targeted area of the neural tube. Therefore, the observed reduction in Cables1 protein indicate a more or less complete removal of Cables1 from the electroporated cells.

Development • Supplementary information

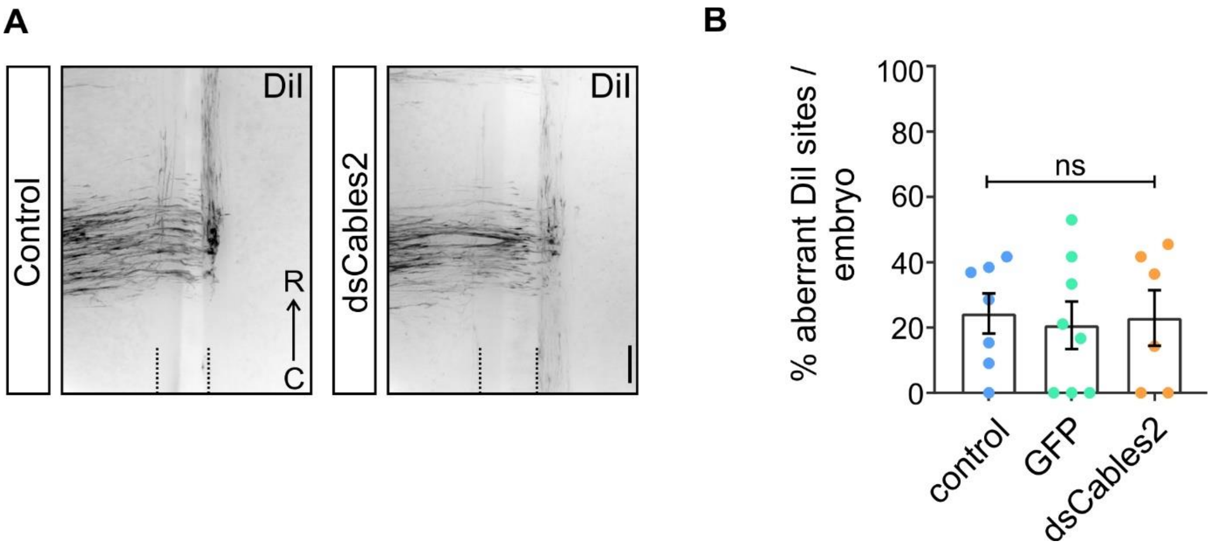

Fig. S3. Downregulation of Cables2 does not impact commissural axon guidance.

Functional analysis of Cables2 revealed no effect on dI1 axon guidance at the floor plate (A). Quantification of aberrant commissural axon trajectories in non-injected control embryos (control, 24.3 ±6.1%; n=92 injection sites in N=7 embryos), GFP-expressing control embryos (GFP; 20.7±7.2%; n=93, N=8), and embryos electroporated with dsRNA derived from Cables2 (dsCables2; 23.0±8.4%; n=55, N=6) showed no significant (ns) difference in the number of DiI injection sites with aberrant axonal trajectories (B). Both untreated and GFP-expressing controls are the same as those shown in Figure 2. Values are given as mean ± s.e.m. One-way ANOVA with Tukey's multiple comparisons test. Scale bar: 50 µm.

Development • Supplementary information

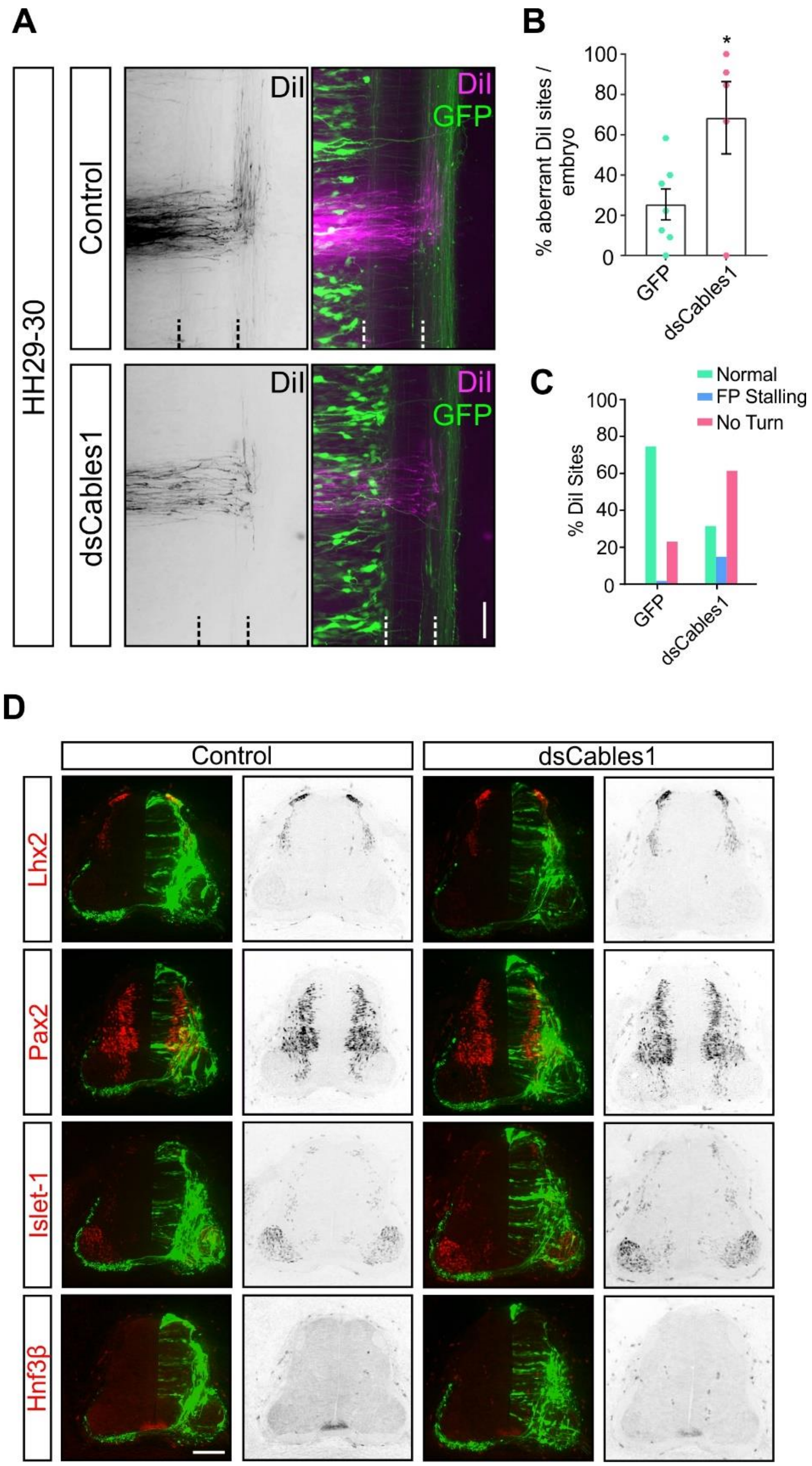

Fig. S4. The axon guidance defects observed after silencing Cables1 are not explained by a reduction in neurite growth speed or due to defects in patterning.

Development • Supplementary information

To exclude a slower growth rate as the reason for the failure of axons to turn into the longitudinal axis in the absence of Cables1, we analyzed dI1 axonal navigation at the floor plate in open-book preparations of spinal cords dissected from embryos at HH29/30, that is 1.5 days older than those shown in Figure 2. The fact that axons still stalled at the floor-plate exit site and failed to turn into the longitudinal axis confirmed that axon guidance defects could not be explained by a slower growth rate, but had to be due to a failure to respond to guidance cues provided by the floor plate (A). (B) When compared to control-treated embryos, aberrant axon guidance was found at 68.4±17.9% of the DiI injection sites in embryos electroporated with dsCables1 (n=56 injection sites in N=5 embryos), compared to control-injected embryos (GFP plasmid only), where aberrant axonal trajectories were seen only at 25.4±7.7% of the injection sites (* p=0.0338; n=74 injection sites in 7 control-injected embryos; unpaired t-test). In contrast to GFP-expressing control embryos, embryos electroporated with dsCables showed an increase in the number of DiI injection sites with axons either stalling in the floor plate, or not turning into the longitudinal axis at the floor-plate exit site (C). Scale bar: 50 µm. Immunostaining of HH25 spinal cord sections with antibodies against Lhx2 (marker for dI1 neurons), Pax2 (interneurons), Islet-1 (motoneurons), and Hnf3β (floor-plate cells) did not reveal any differences in neuronal differentiation between control-treated, GFP-expressing controls, and experimental embryos electroporated with dsRNA derived from Cables1 at HH18 (D). Scale Bar: 100 µm.

Development • Supplementary information

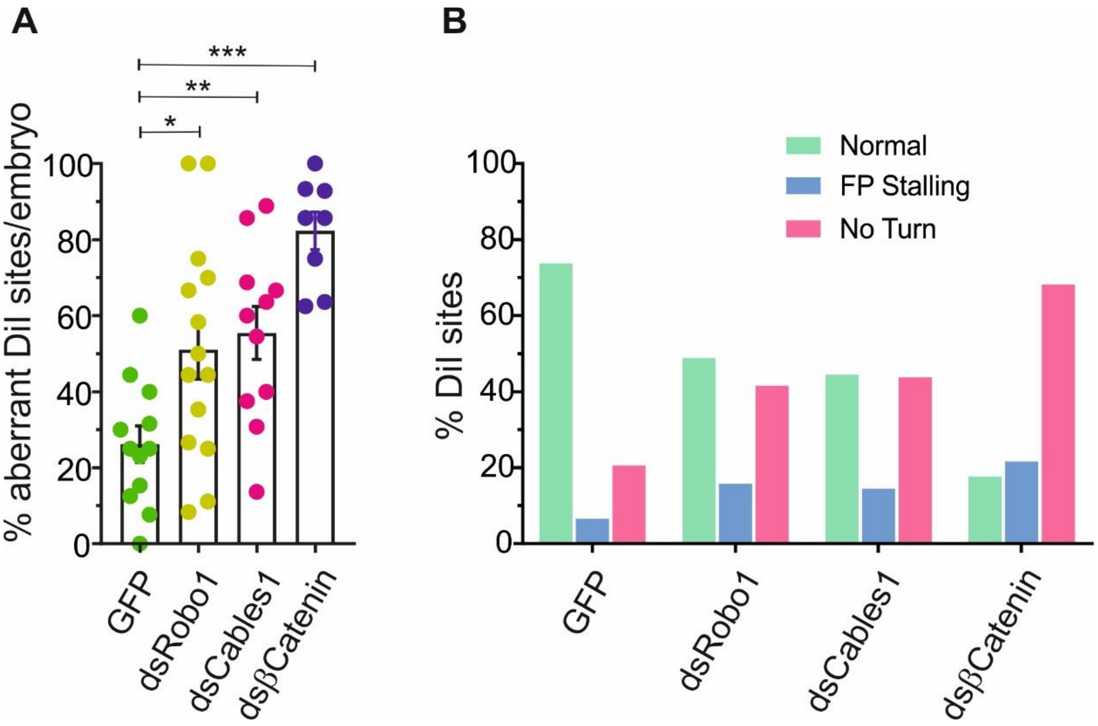

Fig. S5. The dsRNAs derived from *Cables1*, *β-Catenin*, and *Robo1* efficiently perturbed axon guidance when used at regular concentration

Using the regular concentrations of dsRNA derived from Robo1, β-Catenin (300 ng/µl), or Cables1 (500 ng/µl) to silence target genes effectively interfered with commissural axon guidance, as demonstrated earlier (Philipp et al., 2012; Alther et al., 2016; Avilés and Stoeckli, 2016). Knockdown of Robo1 was done by injection and electroporation of dsRobo1 at HH17-18, that is at a time that is not resulting in a maximal effect, as most Robo1 protein is made before that stage and stored in vesicles before being inserted into the growth cone surface by specific trafficking (Alther et al., 2016). However, we wanted to use the same protocol for all groups. Axonal trajectories were aberrant at 51.1±7.8% of the DiI sites in dsRobo1-treated embryos (n=143, N=14), at 55.5±7.0% of the DiI injection sites after silencing Cables1 (n=123 injection sites in N=11 embryos), and at 82.3±4.9% of the injection sites after silencing β-Catenin (n=91, N=8). Embryos injected with the plasmid encoding GFP were used as controls (same group as the one shown in Figure 6). Pathfinding was affected only at 26.2±4.8% of the injection sites in control-treated embryos (n=125 injection sites in N=12 embryos). *p=0.0207, **p=0.0094, ****p<0.0001 ANOVA with Tukey's multiple-comparisons test. (B) Looking at the individual phenotypes indicated that axons were both stalling more often and failed to turn at the exit site in experimental compared to control embryos.

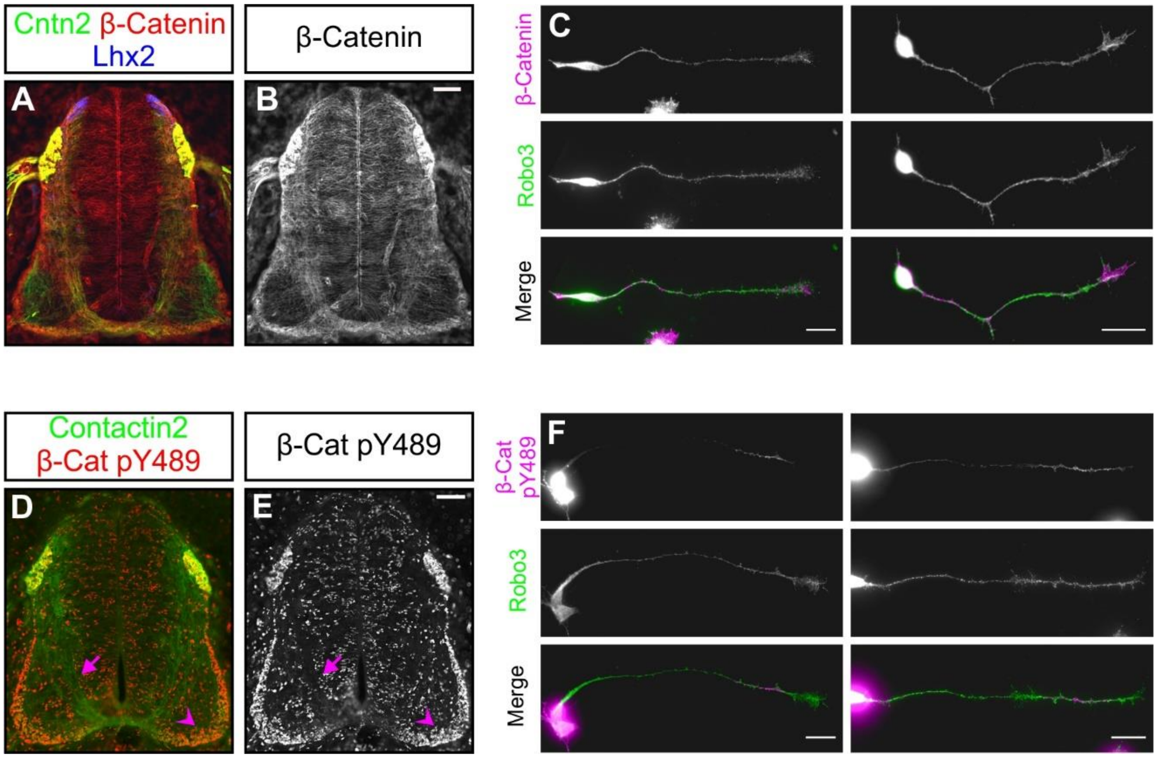

Fig. S6. A phosphorylated form of β-Catenin, β-Catenin pY489, accumulates in distal post-crossing axons.

Transverse sections of spinal cords from HH26 embryos (A, B, D ,E) or dissected neurons (C, F) were stained for total β-Catenin (A, B, C) or β-Catenin pY489 (D, E, F). With an antibody recognizing all forms of β-Catenin, pre- and post-crossing axons were stained (A, B). In contrast, an antibody specific for β-Catenin that is phosphorylated at Y489 revealed higher levels of -β-Catenin pY489 on post-crossing axons (arrowhead), very low levels or no β-Catenin pY489 was found on pre-crossing axons (arrows) (D, E). Commissural axons and axons from dorsal root ganglia (DRG) neurons are visualized with an anti-Contactin 2 (Axonin1) antibody (green, A, D). Staining of cultures of dissociated neurons dissected from embryos sacrificed at HH26 demonstrates the accumulation of β-Catenin pY489 in the distal axon (F), whereas levels of total β-Catenin are more homogenous along the axon (C). Robo3, a marker for dI1 commissural axons is distributed equally along the axon (C, F). Scale bar: 50 µm in A,B,D,E; 20 µm in C,F.

Development • Supplementary information

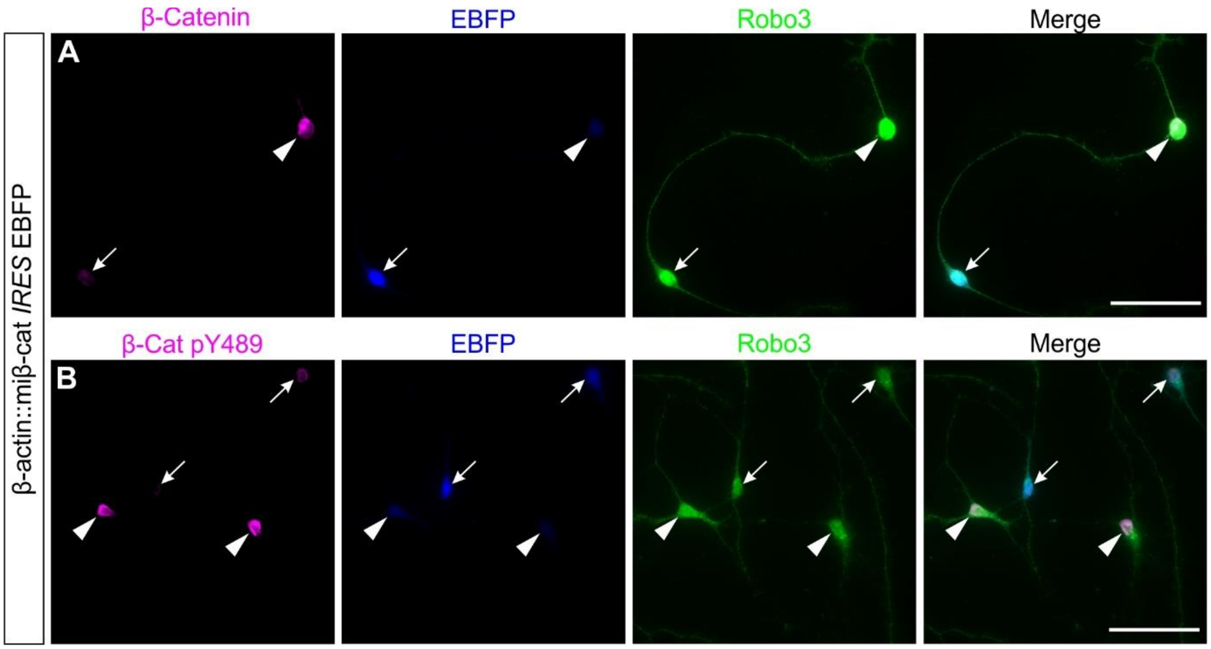

Fig. S7. Loss of β-Catenin in dI1 neurons can be visualized by staining with antibodies recognizing either β-Catenin or β-Catenin pY489.

To verify specificity of our approach and staining of either β-Catenin or β-Catenin pY489 we used a construct expressing a short-hairpin directed against β-Catenin followed by an IRES and blue-fluorescent protein (EBFP). After electroporation at E3, embryos were sacrificed two days later, at HH26, and dI1 neurons were isolated and kept in vitro for 2 days before fixation and staining. Panel A demonstrates that dI1 neurons (green, identified by their expression of Robo3) expressed β-Catenin (magenta; arrowhead). However, those cells that were efficiently targeted by electroporation (indicated by expression of EBFP; arrow) did not exhibit any β-Catenin staining. Similarly, using the monoclonal antibody specific for β-Catenin pY489 (magenta) revealed staining in Robo3-positive dI1 neurons (green) only, if they were not efficiently targeted by the plasmid (arrowhead; compare magenta staining in panel B with blue staining). Those that were efficiently targeted (arrows) did not show any β-Catenin pY489 staining. Bar: 50 µm.

Development • Supplementary information

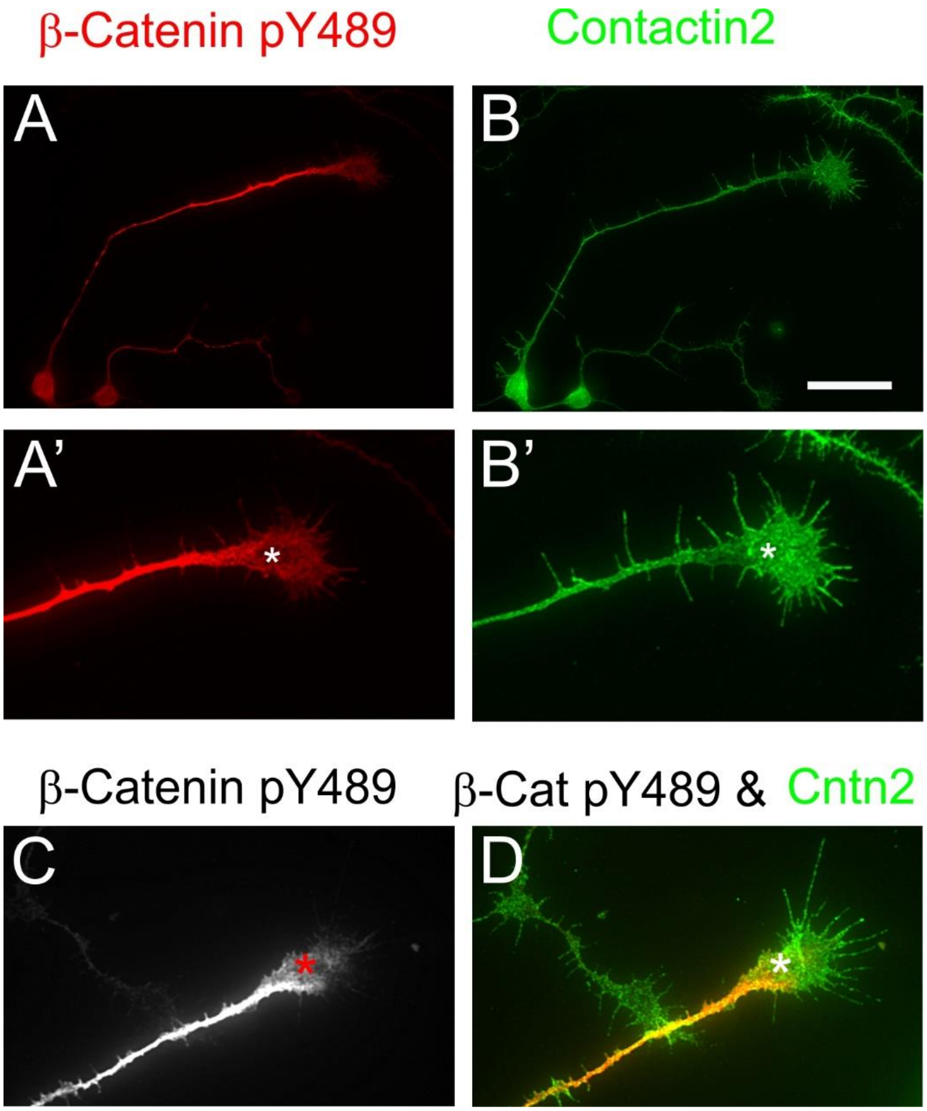

Fig. S8. β-Catenin pY489 localizes predominantly to the distal axon, the transition zone to the growth cone and in the central part of the growth cone.

In cultured post-crossing commissural axons, β-Catenin pY489 was found at higher levels in the distal axon (A; see also Figures 7 and 8). Contactin2 was used to stain axons and growth cones of dI1 neurons (B). β-Catenin pY489 is localized in the central part of the growth cone (A'), but not found in filopodia (compare staining for Contactin2 in B'). (C,D) Another example of a growth cone stained for β-Catenin pY489 (C) and shown in combination with Contactin2 staining (D, β-Catenin pY489 in red, Contactin2 in green). Bar: 20 µm in A,B; 10 µm in A',B',C,D.

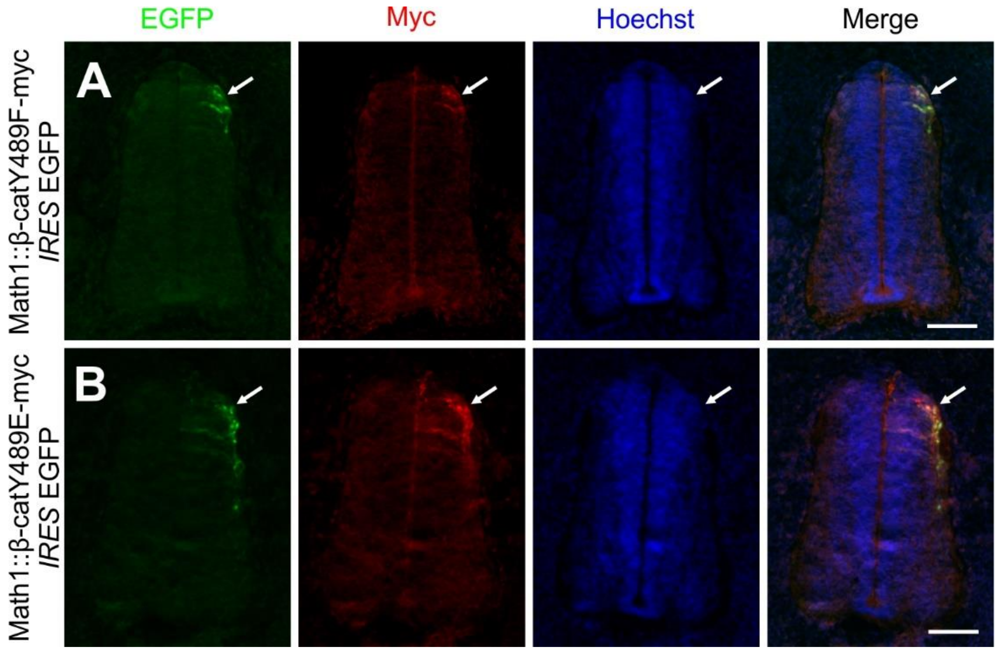

Fig. S9. Math1 drives expression of mutant version of β-Catenin.

Both the dominant negative version of β-Catenin, β-Catenin Y489F (A), which cannot be phosphorylated, and the dominant active version of β-Catenin, β-Catenin Y489E (B), are expressed in dI1 neurons when controlled by the Math1 enhancer. Electroporated constructs contained the myc-tagged version of the respective β-Catenin (either Y489F or Y489E) followed by an IRES site and EGFP. Hoechst staining shows nuclei.

Table S1. Reagents and resources

REAGENT or RESOURCE	SOURCE	IDENTIFIER
**Antibodies**		
Rabbit anti-Cables1, 1:1000 IF	ABCAM	Cat#ab130967 (Discontinued)
Rabbit anti-Cables1, 1:750 WB	Origene	AP06793PU-N
Rabbit anti-GAPDH, 1:2500 WB	ABCAM	Cat#ab9485
Mouse IgM anti-β-Catenin pY489, Supernatant 1:100 IF	Hybridoma Bank	
Rabbit anti-β-Catenin, 1:500 IF	Santa Cruz	Cat#Sc-7199 (Discontinued)
Mouse anti-Lhx2, 1:50 IF	Developmental Studies Hybridoma Bank	PCRP-LHX2-1C11
Mouse anti-Hnf3β, 1:2 IF	Developmental Studies Hybridoma Bank	4C7
Mouse anti-Neurofilament, 1:150 IF	Invitrogen	#RMO-270
Mouse anti-Islet1, 1:10 IF	Developmental Studies Hybridoma Bank	40.2D6
Rabbit anti-Pax2, 1:200 IF	Invitrogen	#71-6000
Rabbit anti-RFP, 1:2000 IF	Antibodies Online	Cat#ABIN129578
Goat anti-Robo3, 1:500 IF	R&D Systems	Cat#AF3076
Rabbit anti-Axonin1/Contactin2, 1:500	Stoeckli and Landmesser, 1995	
Goat anti-GFP-FITC, 1:500 IF	Rockland	Cat#600-102-215
Donkey anti-mouse Cy3, 1:2000 IF	Jackson Immuno Research	Cat#715-165-150
Goat anti-mouse Cy3, 1:2000 IF	Jackson Immuno Research	Cat#115-165-003
Goat anti-mouse IgM TRITC	Cappel	Cat# 55531
Donkey anti-rabbit Cy3, 1:2000 IF	Jackson Immuno Research	Cat#711-165-152
Donkey anti-rabbit Alexa Fluor 488, 1:1000	Invitrogen	Cat#A21206
Anti-DIG-AP, Fab 1:1000 ISH	Roche Diagnostic	Cat#11093274910
Goat anti-rabbit HRP	Jackson Immuno Research	Cat#111-035-003
**Chemicals, peptides, and recombinant proteins**		
Fast DiI	Thermo Fisher Scientific	Cat#D7756
NBT	Axonlab	Cat#A1117,0001
BCIP	Axonlab	Cat#A1243,0001
mSlit2 CF	R&D Systems	Cat#5444-SL
hWnt5a	R&D Systems	Cat#645-WN-010
RhNetrin1 CF	R&D Systems	Cat#6419-N1
PBS pH 7.4	Life Technologies	Cat#10010056
Mowiol 4-88 Reagent	Merck	Cat#475904

Development • Supplementary information

Trypsin EDTA 10X	Invitrogen	Cat#15400054
DIG RNA labeling mix	Roche Diagnostic	Cat#11277073910
T7 RNA polymerase	Promega	Cat#P207B
SP6 RNA polymerase	Promega	Cat#P108B
ECL Western Blotting Detection Reagent	GE Healthcare	RPN2209
Q5 High-Fidelity DNA Polymerase	New England Biolabs	Cat#M0491L
**Commercial assays**		
Q5 Site-Directed Mutagenesis Kit	New England Biolabs	Cat#E0554S
NEBuilder® HiFi DNA Assembly Master Mix	New England Biolabs	Cat#E2621S
SuperScript™ III First-Strand Synthesis SuperMix	Thermo Fisher Scientific	Cat#18080-400
Fast Sybr Green Master Mix	Thermo Fisher Scientific	Cat#4385610
**Experimental models: organisms/strains**		
Gallus gallus: Hubbard JA57 strain	Brüterei Stöckli, Ohmstal	N/A
**Oligonucleotides**		
qPCR chicken *Cables1_X1*-specific primer sequence, forward: TACCCAAGTCGGGGACATGA	Microsynth AG	N/A
qPCR chicken *Cables1_X1*-specific primer sequence, reverse: CGAGTTCCGAGAGCATTGGT	Microsynth AG	N/A
qPCR chicken *Cables1_X2*-specific primer sequence, forward: GGAAAATGCCCCACTACGCA	Microsynth AG	N/A
qPCR chicken *Cables1_X2*-specific primer sequence, reverse: CCAACTTCATGTCCCTGCCAT	Microsynth AG	N/A
qPCR chicken *Cables1_X3*-specific primer sequence, forward: CCCACATTCCCCATTCGCC	Microsynth AG	N/A
qPCR chicken *Cables1_X3*-specific primer sequence, reverse: GGGAGATCAGCCGACGTCTATG	Microsynth AG	N/A
qPCR chicken *Cables2_X1*-specific primer sequence, forward: AGAGAAGGCGTTTTATCTCCCAG	Microsynth AG	N/A
qPCR chicken *Cables2_X1*-specific primer sequence, reverse: GCGCAGATTAATACGATCCTGCTG	Microsynth AG	N/A
qPCR chicken *18S*-specific primer sequence, forward: CGAAAGCATTTGCCAAGAAT	Himmels et al.,2017	
qPCR chicken *18S*-specific primer sequence, reverse: GGCATCGTTTATGGTCGG	Himmels et al.,2017	
**Recombinant DNA**		
ChEST446e14 (Cables1)	Source BioScience	
ChEST809a5 (Cables 2)	Source BioScience	
ChEST822o14 (Cables 1)	Source BioScience	
ChEST231k15 (β-Catenin)	Source BioScience	
CAG::HA-hRobo1-myc	Philipp et al., 2012	
CAG::mRuby3-mCables	This paper	
β-actin::EGFP-F	Baeriswyl et al., 2021	

Development • Supplementary information

β-actin::miβ-Catenin_IRES-EBFP	Avilés and Stoeckli, 2016	
Math1::tdTomato-F	Wilson and Stoeckli, 2013	
Math1::EGFP-F	Wilson and Stoeckli, 2013	
pCAGGs::hrGFP	Wilson and Stoeckli, 2011	
Math1::mCables1-myc_IRES-EGFP	This paper	
CAG::mβ-Catenin-myc_IRES-EGFP	This paper	
Math1::mβ-CateninY489E-myc_IRES-EGFP	This paper	
Math1::mβ-CateninY489F-myc_IRES_EGFP	This paper	
**Software**		
ImageJ	Schneider et al., 2012	https://imagej.nih.gov/ij/
Prism 8	GraphPad	https://www.graphpad.com/scientific-software/prism/;%20RRID:%20SCR_002798/

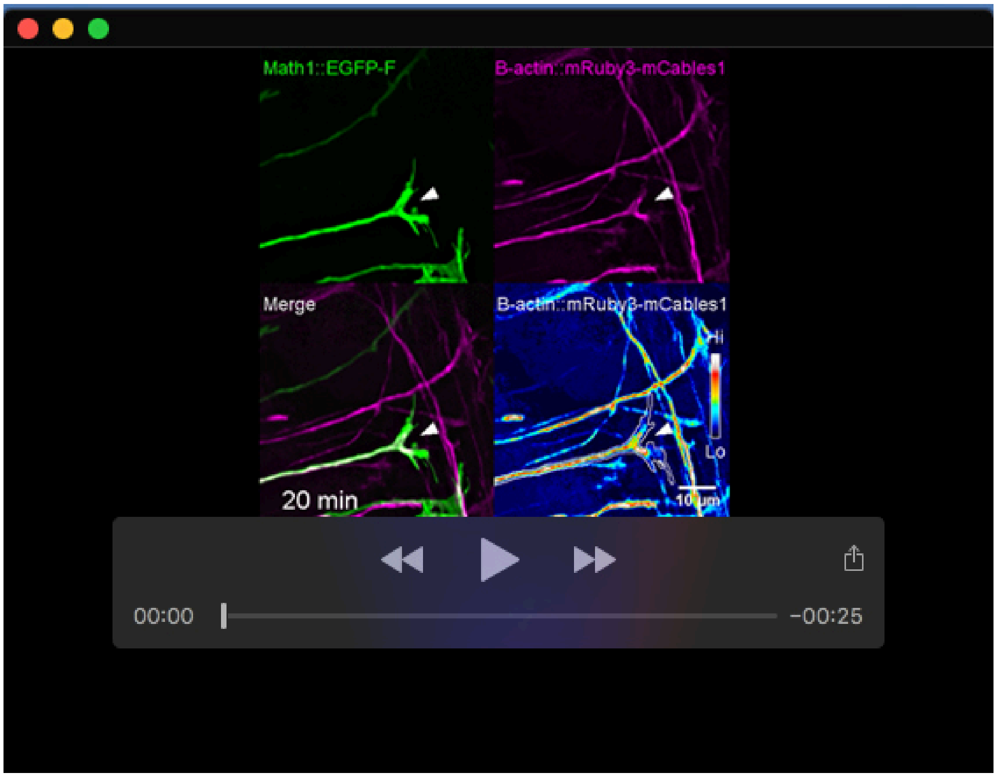

Movie 1. mRuby3-mCables1 localizes to dI1 growth cones at the floor-plate exit site.

Mouse Cables1 (mCables1) was fused to mRuby3 (shown in magenta) and electroporated *in ovo* into commissural neurons together with a Math1::EGFP-F plasmid to specifically label dI1 neurons (shown in green). This video shows a dI1 growth cone exiting the floor plate and turning rostrally (white arrowhead) in an intact cultured spinal cord using live imaging (see Figue 3A). In the lower right panel a heat map of pseudo-colored mRuby3-mCables together with the edge of the growth cone traced as a white line are shown. A clear signal of mRuby3-mCables could be seen in this growth cone during the exit of the floor plate as well as the rostral turn (white arrowhead). One stack was taken every 10 min for 90 min. Hi, high; Lo, low. Rostral is up.

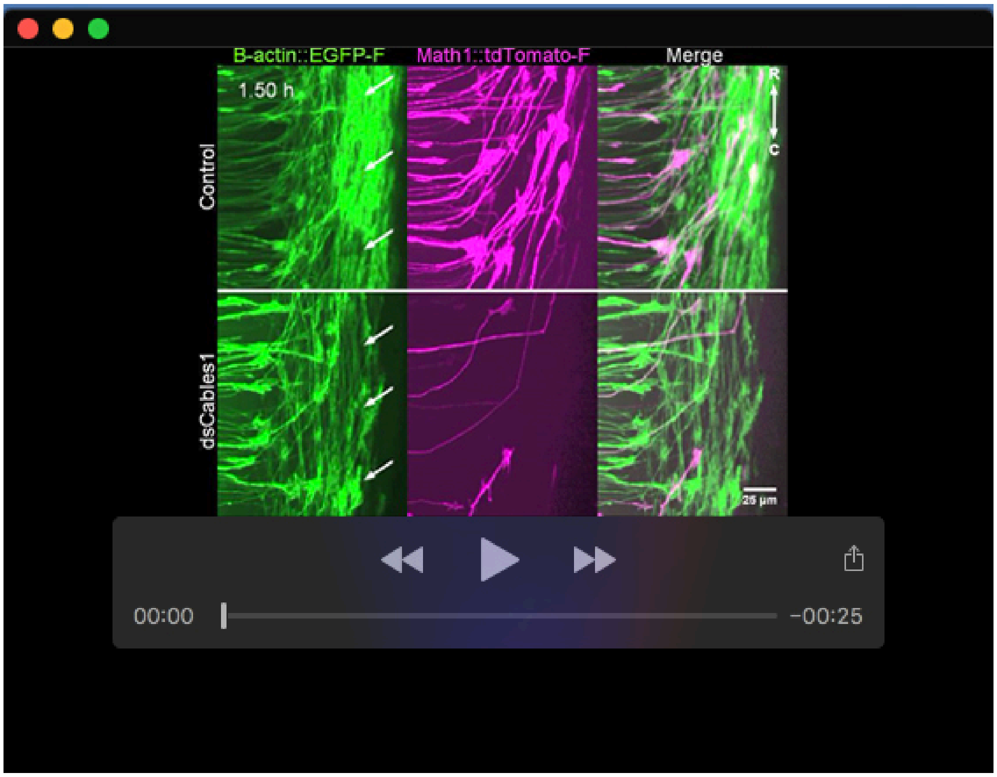

Movie 2. Twenty-four-hour time-lapse recording of dI1 axons crossing the floor plate in cultured intact spinal cords.

dI1 neurons were visualized by *in ovo* electroporation of the Math1::tdTomato-F plasmid (shown in magenta). In the control condition, dI1 axons turned rostrally in a well-organized manner after exiting the floor plate. However, knockdown of Cables1 induced aberrant phenotypes at the floor-plate exit site with axons turning caudally (yellow arrow) or having problems to extend rostrally (yellow arrowhead). These aberrant phenotypes clearly induced the formation of a disorganized ventral axon bundle as shown by all commissural axons expressing EGFP-F (green) compared to the tightly organized control funiculus (white arrows). R, rostral; C, caudal.

Development • Supplementary information
