# Supplementary material for: Cables1 links Slit/Robo and Wnt/Frizzled signaling in commissural axon guidance
Source: Development. 2023 Oct 9;150(19):dev201671. doi: 10.1242/dev.201671 (PMC10617602; doi:10.1242/dev.201671)
Supplement: Supplementary file 1 [file develop-150-201671_review_history.pdf]

Cables1 links Slit/Robo and Wnt/Frizzled signaling in commissural axon guidance

Esther Stoeckli, Nikole Zuniga, Alexandre Dumoulin and Giuseppe Vaccaro
DOI: 10.1242/dev.201671

Editor: James Briscoe

Review timeline

Submission to Review Commons:	15 October 2021
Submission to Development:	31 January 2023
Editorial decision:	13 March 2023
First revision received:	1 August 2023
Accepted:	7 September 2023

Reviewer 1

Evidence, reproducibility and clarity

In this work by Zuñiga et al. the authors study the role of the adaptor protein Cables1 on the guidance of post-comissural spinal cord neurons. They hypothetize that commissural axons need Cables1 to leave the floor plate and turn to ascend to the brain. They propose that during this process, Cables1 acts as a linker of two key axon guidance pathways, Slit and Wnt. Cables1 would localize β-catenin phosphorylated at tyrosine 489 to the distal axon and this would be necessary for the correct turning and navigation of post-crossing commissural axons. Although the work may be potentially interesting, there are major issues that authors need to address in order to state their claims:

-Fig. 2. To visualize the axonal phenotype after downregulation of Cables1 the authors use DiI labelling. This difficults the interpretation of the results as both electroporated and non-electroporated axons are labelled. Since the authors have a Math1::tdTomatoF reporter construct (as in Fig. 3), it would be desirable to use this construct Math1::tdTomatoF in combination with the dsCables1 plasmid to better visualize the phenotype. Alternatively and less preferred, GFP signal should be also shown in Fig.2B experiments.

-Fig. 2B and Supp.Fig.3. Comparable DiI labellings should be shown in the different conditions. The three examples shown in this panel despite different amount of DiI-labeled axons making it difficult to compare them.

-Fig. 2D. An scheme depicting the different phenotypes: "normal", "FP stalling" and "no turn" would help to understand the results. They can use schemes similar to those shown in Fig. 2K Parra et al. 2010.

-Fig. 3A. The open-book drawing is confusing. It seems that they are analyzing open-book preparations in this experiment when this is not the case.

-Fig. 3B. Authors claim that Cables1 is not required in pre-crossing axons as dsCables electroporation does not affect axonal growth of DiI neurons taken at HH22. However, to be sure that Cables1 mRNA levels are downregulated in pre-crossing axons, relative levels of Cables1 mRNA and/or protein should be also determined at HH22 not only at HH25.

-Fig. 4. The incapacity of Slit to induce axonal retraction in dsCables1 neurons is used to conclude that Cables1 is required to respond to Slit. However, downregulation of Cables1 by itself is even

© 2023. Published by The Company of Biologists under the terms of the Creative Commons Attribution License (https://creativecommons.org/licenses/by/4.0/).

more effective inhibiting axonal growth than Slit treatment. Upon this strong effect as a background, it is difficult to assay slit response. Authors should point this observation in the manuscript.

-Fig. 5B. In this Figure they do not differentiate between FP stalling or no turn phenotypes. A quantification taking into account the different phenotypes as shown in Fig.2D should be included.

-Fig. 6D,E. As postulated in the manuscript and based on the Rhee, et al. paper, the β-catenin phosphorylation is triggered by Abl quinase upon Slit-Robo signaling. How the authors explain then that isolated cells with axons growing on a plate recapitulate specific distal phosphorilation of β-catenin at Y489 in the absence of Slit signaling? This experiment shows that postcrossing axons contain more phosphorylated β-catenin as an intrinsic characteristic rather than as a consecuence of contact with floor plate signals. Authors should try a similar experiment but exposing the neurons (or explants) to Slit. Also, why β-catenin phosphorylation was not measured at the growth cone?

-Fig. 7. CAG::hrGFP electroporation is not specific for dl1 neurons. This experiment should be performed with Math1::tdTomatoF in order to analyze β-cat pY489 with or without dsCables1 specifically in dl1 neurons. Also, why GFP staining at the growth cones in Fig.7B is not visible in the axon?

-Fig. 8. This experiment does not distinguish whether phosphorylated β-Cat is necessary for the correct navigation of post-crossing commissural axons (as it is claimed in the abstract) or it is also required for midline crossing. As it has been previously shown, correct navigation of post-crossing commisusal axons is a Wnt5 dependent process. As dsCables1 abrogates Wnt5a responsiveness (Fig. 4B,C), does the phosphomimetic β-catenin Y489E construc rescue the Wnt5a response in dsCables1 electroporated neurons? Moreover, can the phosphomimetic β-catenin Y489E construc rescue the Slit response in dsCables1 electroporated neurons? Testing these effects on explants as in Fig. 4B,C but including phosphomimetic β-catenin, will help to understand to what extend phosphorylation of β-catenin is important for crossing, turning or both processes.

-How do the authors envision the mechanism of Cables1/β-catenin mediated crossing and turning? A working model summarizing their hypothesis would help the reader to understand the results.

Minor points:

-Homogeneize the term "scale bars" or "bars" in the Figure Legends.

-Scale bar of insets in Fig.1C is missing.

-The antisense control for Cables probe should be shown at HH-22/24. Otherwise is not possible to distinguish whether they do not detect signal because is a negative control or because Cables1 is not expressed at HH25.

-Figure legend for Fig. 2D is missing

-Fig. 8B right panel is contaminated with growthing axons coming from the below DiI injection. Please replace the picture.

-The quantification of the different phenotypes "FP stalling", "no turn" should be better explained in the Mat and Met section. The sentence " more than 50% of the axons...." is not clear. Was this measured by eye? Otherwise, please indicate the software used to measure.

-Provide the quantification of the WB in Supplementary Fig. 2B normalising to Gapdh.

© 2023. Published by The Company of Biologists under the terms of the Creative Commons Attribution License (https://creativecommons.org/licenses/by/4.0/).

Significance

Previous results have demonstrated that Slit-induced modulation of adhesion is mediated by cables that links Robo-bound Abl kinase to N-cadherin-bound betacat (Rhee et al., 2007). Here the authors propose that a similar mechanism is operating in commissural neurons leave the midline after crossing and turn immediately after. The role of Cables in the process has not been previously addressed. Thus, after proper addressing of my main concerns, I consider this paper may advance in our knowleged of how growing axons navigate intermediate targets.

Reviewer 2

Evidence, reproducibility and clarity

In this paper entitled "Cables1 links Slit/Robo and Wnt/Frizzled signaling in commissural axon guidance", authors aim to the find the mechanisms the coordinate the floor plate exit and the rostral turning of commissural axons.

During development thousands of axons have to navigate long distances to reach their targets and build functional circuits. To facilitate their journey, their paths is divided into small portions by intermediated targets. The most studied intermediated target is the floorplate (FP) at the midline of the ventral spinal cord. Glia cells forming the FP express plethora of guidance cues. Commissural neurons, which have their cells bodies located in the dorsal part of the spinal cord, send their axons towards the FP. These axons are first attracted by the FP which facilitate their entry within the FP. However, they switch this attractive response into a repulsive one in order to exit the FP and turn rostrally to connect their brain targets.

In order to ensure that this process will go smoothly, commissural axons have to adapt the composition of their receptors and the signaling pathways to switch from attractiveness to repulsion.
So far, many processes have been involved such as the alternative splicing of receptors (Robo3; Chen et al Neuron 2008), protease regulation of receptor expression (Nawabi et al Genes & Dev 2010), trafficking of receptors, or their interaction profiles (Delloye et al Nat Neuro 2015). However, it is still not clear how 2 events (here exit from the FP and rostral turning) are linked.

Authors propose an original mechanism that involved the adaptor protein Cables 1. This protein has been shown to link the Robo/Slit1 signaling to Cadherins. Cables regulates the repulsive response to Slit and adhesion by the phosphorylation of b-Catenin by the kinase Abelson (Rhee et al Nat Cell Biol 2007).

The story developed here is very original and interesting: Cables would link the exit of FP (mediated by Robo/Slit signaling) and the rostral turning of the commissural axons (controlled by the Wnt/Fzd pathway. Below I'm proposing some experiments as many questions raised upon reading this beautiful work. The experiments are sound and could be reproducible. The statistic analysis looks fine.

I would suggest some experiments to strengthen the whole work:

•Authors might want to consider to perform some biochemistry experiments to show that Cables is able to interact with Robo1 and Fzd3: are these proteins in the same molecular complex? They could do 2 experiments: one in vitro by transfecting a cell line (such as HEK293 or cos cells) with plasmids coding for Robo1, Cables and Fzd3 or at least Cables and Fzd3 (as for Robo1/Cables they could refers to Rhee et al 2007). Another one would be in vivo: extracting proteins from the pre-crossing stage, the FP and post crossing stage; immunoprecipitation of Cables1 and see whether Robo1 and/or Fzd are pull down with Cables 1.

•From the pictures it seems that most of the axons are stalling in the FP when embryos are electroporated with dsCables1. It would be nice to show more examples of axons that are able to exit the FP but have turning problems. Given the data, as it is presented, it seems that Cables regulates more the FP exit (and therefore, as it was shown in Rhee et al, the responsiveness to

© 2023. Published by The Company of Biologists under the terms of the Creative Commons Attribution License (https://creativecommons.org/licenses/by/4.0/).

Robo/Slit signaling). In the same line, in Fig 4, Authors need to add a condition using dsCables and ds Fzd in order to see the effect of Cables on axon turning (response to Wnt). As it is this figure supports the role of Cables on FP exit but it's hard to make the link with commissural axon responsiveness to Wnt.

•Authors aim to show that Cables is a linker between 2 events: maybe it should be nice to try to disconnect these events. One way would be (if technically possible) to modulated expression of Cables at different stages. What would happen if Cables was down regulated upon FP crossing? Would axons still be able to respond to Wnt? The question I'm wondering about is whether the responsiveness to Slit and Wnt is acquired at the same time or whether axons should become sensitive to Slit and this event will prime them to respond to Slit. In order to address the following experiment could be performed: explants from HH22-HH23 embryos, could be treated with medium containing Slit first and then Wnt or vice et versa and perform some collapse assay.

•In Fig3 I was wondering whether post crossing axons were growing less because of the change in the regulation of adhesion: Rhee et al shows that Cables is able to modulate adhesion through N-cadherin. It would be interesting to perform immunostaining on these explant cultures to assess any change in adhesion molecules.

•It is not clear whether Robo1 and/or Fzd induces the phosphorylation of b-catenin: is the Robo1/Slit binding induce the phosphorylation of b-cat and this event will prime the axons to respond to Wnt/Fzd? Or Wnt/Fzd is also able to control b-cat phosphorylation?

•The staining with the antibody needs to be detailed: as it is reported this antibody recognizes "a domain of Cables1 that is 90% identical to the corresponding region of Cables2": it seems that the Cables protein enrichment in the floor plate (around the central canal) is Cables 2 as its mRNA expression matches this profile of expression. The one expressed in the crossing axons might be Cables 1: one way to verify this, is to perform the staining on sections from embryos electroporated with dsCables 1. This is a very important control of the antibody to reinforce this point of the paper.

•In Figures 3-4: why not performing some co culture of spinal cord explants with COS or HEK 293 cells expressing Slit1 or Wnt? This experiment will provide a clear-cut response to see the role of Cables in axon guidance. As there it is, Fig3 shows a role of Cables in axon growth but not guidance.

•In Figure 6: my understanding of axon guidance is that every guidance decision happens at the level of the growth cone. However, it seems that in post crossing stage, there is a strong decrease of b-cat and phosphor- b cat within the growth cone compared to the precrossing stage. If beta cat is the effector of Cables to link Robo/Slit and Wnt/Fzd signaling I would expect it to be localized at the growth cone. I think authors should discuss this point. Regarding the normalization, it would be better to counterstaing the neurons with actin and use the measure of its fluorescence to normalize phopho-beta cat.

Minor points:

•In figure 2: it seems that there are few axons labelled with DiI in the dsCables1 condition (Fig2B): it would be the choice of the picture or maybe the downregulation of Cables 1 interfere with the survival of dI1 neurons (even though in supp 1C it is shown that most of the populations are still there with no difference with the control side) or maybe some axons are delayed to reach to FP on time: the picture is focused on the FP: are there any axons still growing in the side of the open book preparation? Again, the picture that could be misleading.

•In Fig1 legends, maybe Authors wanted to write "At HH18 dI1 commissural neurons start to extend their axons in the ventral spinal cord"?

•I would also remove the yellow shadow on the Fig1A: it could be misleading as at first glance the reader might wonder whether there are 2 populations of dI1 neurons.

© 2023. Published by The Company of Biologists under the terms of the Creative Commons Attribution License (https://creativecommons.org/licenses/by/4.0/).

Significance

It is still not clear how axons cross the midline. So far, many processes have been involved such as the alternative splicing of receptors (Robo3; Chen et al Neuron 2008), protease regulation of receptor expression (Nawabi et al Genes & Dev 2010), trafficking of receptors, or their interaction profiles (Delloye et al Nat Neuro 2015). However, it is still not clear how 2 events (here exit from the FP and rostral turning) are linked.

Authors propose an original mechanism that involved the adaptor protein Cables 1. This protein has been shown to link the Robo/Slit1 signaling to Cadherins. Cables regulates the repulsive response to Slit and adhesion by the phosphorylation of b-Catenin by the kinase Abelson (Rhee et al Nat Cell Biol 2007).

The audience that will be interested in this work is the neurodevelopment filed, axon regeneration field and overall people interested in neuronal circuit formation and function.

My field of expertise is molecular and cellular neuroscience applied to axon guidance (crossing the FP) in mice models, axon regeneration and circuit formation.

Reviewer 3

Evidence, reproducibility and clarity

In their manuscript, Zuniga and Stoeckli characterize the role of Cables in commissural axon guidance in the developing chick spinal cord. Based on a combination of in vitro outgrowth assays and in vivo dye-tracing experiments, the authors propose that Cables participates in both normal repulsive responses to Slit and attractive responses to Wnt5. Using combinations of low-does knock down of cables/robo and or B-catenin, the author suggest an in vivo link between these pathways. Using IF with phospho-specific antibodies to B-catenin, the authors suggest that there is elevated P-Bcatenin in the post-crossing segments of distal axons. While potentially interesting, the present study falls short of adequately supporting the major claims. In addition, there are several instances where experiments lack appropriate controls.

Specific Comments

The conclusions reached by the authors are over-stated given the experiments performed. For example, the authors describe 'silencing' cables throughout the paper; however, the knock down that they achieve is approximately 50%. Indeed, it is quite surprising that such strong effects on growth/guidance can be achieved with a two-fold depletion of the gene product. Nevertheless, the rescue experiments provide nice evidence that dsRNA for Cables is causing a phenotype.

This partial knockdown precludes strong conclusions, like for Figure 3, where they state that 'Cables is not required for pre-crossing.' The language needs to be tempered.

Figure 4: the authors use bath application of Slit and Wnt to test effects of cables on Slit and Wnt responses. The observed effect sizes are very small and a single assay of this type does not allow such strong conclusions like 'loss of Cables prevents responsiveness.' Again, it is difficult to imagine that 50% reduction would completely prevent responses, raising questions about the suitability of this assay for measuring responsiveness- perhaps growth cone collapse would give more convincing results.

Figure 5: The authors should more clearly document the effects they are seeing in these manipulations. As written, all we know is that there are 'significant effects on axon guidance.' What are these effects? Do they see the predicted differences between robo/cables and Bcatenin/cables phenotypes? e.g re-crossing defects in the case of robo and anterior turning defects in the case of B-catenin?

Also related to Figure 5:

© 2023. Published by The Company of Biologists under the terms of the Creative Commons Attribution License (https://creativecommons.org/licenses/by/4.0/).

The authors do not validate the dsRNA knockdown of either Robo or B catenin.
It is unclear what the interpretation or expectation of the triple knock down condition is.

Figure 6: For this reviewer images showing enhanced P-Catenin in post-crossing distal axons is not convincing. The differences are not obvious by eye and the quantification suggests an ~30% increase. In contrast a nearly 4-fold increase is reported in Figure 7 for this same measurement. This raises concerns about the reproducibility of this 'phenotype.'

Also related to Figure 6:

No validation of antibody specificity is provided or described.

Figure 8: As for figure 5, phenotypic documentation is incomplete. In addition, no controls are shown to assure that the different mutant forms of B-catenin are comparably expressed, nor is there an unmutated wild-type control. The authors state that expression of these constructs alone has no effect on normal guidance; however, the supplemental data 6B would seem to indicate that both forms lead to increases abnormal phenotypes.

Significance

The work builds on in vitro observations from Rhee, 2007 about links between Robo signaling and Cables function. If adequately demonstrated, integration and coordination of Robo and Wnt axon guidance pathways is quite significant.

Author response to reviewers' comments

[Please use this template only if the submitted manuscript should be considered by the affiliate journal as a full revision in response to the points raised by the reviewers.

If you wish to submit a preliminary revision with a revision plan, please use our "Revision Plan" template. ***It is important to use the appropriate template to clearly inform the editors of your intentions.]***

1. General Statements [optional]

Dear editors at Review Commons

Thanks for your patience. We have finally carried out a full revision of our originally submitted manuscript summarizing our findings on the role of Cables1 in axon guidance.
In our study, we provide in vitro and in vivo evidence for a role of Cables1 as a linker between axon guidance signaling pathways. Commissural axons in the developing spinal cord leave their intermediate target, the floor plate, due to a switch from attraction to repulsion mediated by the specific trafficking of Robo1 receptors to the growth cone surface. The presence of Robo1 on growth cones after contact with the floor plate allows them to respond to Slit, the negative guidance cue associated with the floor plate. After leaving the floor plate on the contralateral side, growth cones respond to a Wnt gradient along the antero-posterior axis. The responsiveness to Wnt of post- but not pre-crossing axons is regulated by the trafficking of Fzd3 receptors to the growth cone membrane of post-crossing axons (Alther et al., 2016), but also by the specific phosphorylation of β-Catenin at tyrosine Y489 by Abl kinase. Cables1 mediates this phosphorylation by transferring Abl kinase from the C-terminus of Robo1 to β-Catenin (this study).

The revised version of the manuscript contains additional experiments in vitro, in vivo and ex vivo combined with live imaging to further support our conclusion about the role of Cables1 as a linker between Robo/Slit and Wnt signaling.

© 2023. Published by The Company of Biologists under the terms of the Creative Commons Attribution License (https://creativecommons.org/licenses/by/4.0/).

It took as longer than expected to carry out these new experiments, as Nikole Zuñiga, the first author of the paper, left the lab after her PhD defense to take up a job in industry. Unfortunately for the study, but fortunately for Giuseppe Vaccaro, he also got a job soon after taking over the project. Therefore, the revision was delayed again. We hope that the additional experiments will solve the issues that were raised by the reviewers. We thank them for their contributions and suggestions.

Best regards
Esther Stoeckli

2. Point-by-point description of the revisions

This section is mandatory. Please insert a point-by-point reply describing the revisions that were *<u>already carried out and included</u>* *in the transferred manuscript.*

Point to point response to reviewers' comments

Reviewer #1 (Evidence, reproducibility and clarity (Required)):
In this work by Zuñiga et al. the authors study the role of the adaptor protein Cables1 on the guidance of post-comissural spinal cord neurons. They hypothesize that commissural axons need Cables1 to leave the floor plate and turn to ascend to the brain. They propose that during this process, Cables1 acts as a linker of two key axon guidance pathways, Slit and Wnt. Cables1 would localize β-catenin phosphorylated at tyrosine 489 to the distal axon and this would be necessary for the correct turning and navigation of post-crossing commissural axons. Although the work may be potentially interesting, there are major issues that authors need to address in order to state their claims:

-Fig. 2. To visualize the axonal phenotype after downregulation of Cables1 the authors use DiI labelling. This difficults the interpretation of the results as both electroporated and non-electroporated axons are labelled. Since the authors have a Math1::tdTomatoF reporter construct (as in Fig. 3), it would be desirable to use this construct Math1::tdTomatoF in combination with the dsCables1 plasmid to better visualize the phenotype. Alternatively and less preferred, GFP signal should be also shown in Fig.2B experiments.

We respectfully disagree. Most likely, the reviewer thinks about a defined nerve that has a particular trajectory and then when labelled with a fluorescent marker, deviations from this pathway, or defasciculated growth can be easily visualized. However, in the spinal cord, the dI1 axons run ventrally more like a 'curtain'. Therefore, the aberrant behavior of axons is difficult to see. We therefore, opted for the alternative suggestion and added the GFP images to visualize clearly that the axons labelled with DiI are from the injected area. We also would like to add that we are extremely careful in injecting DiI only to the dI1 population of commissural axons to avoid mixing populations with different trajectories. As the analysis is done by a person blind to the experimental condition, we are convinced that our way of analyzing the phenotype is valid. An approach that has been successfully used by many groups for decades now. Please also keep in mind that we are always comparing groups of embryos with each other. Furthermore, having axons traced by DiI which were not targeted by dsRNA electroporation would not increase but rather decrease the likelihood of aberrant behavior. Therefore, we are convinced that our method of quantification is valid.

However, we have added new experiments using live-imaging which also demonstrate that many axons in embryos electroporated with dsCables1 fail to turn properly at the floor-plate exit site (see Movie 2). These experiments provide additional evidence for the validity of our results.

-Fig. 2B and Supp.Fig.3. Comparable DiI labellings should be shown in the different conditions. The three examples shown in this panel despite different amount of DiI-labeled axons making it difficult to compare them.

We have exchanged the image of the control-treated embryo in Figure 2 to have more comparable DiI injection sites. However, as we detail in our Material & Method section, the quantification was done in such a way that the number of axons does not matter. We rephrased this paragraph to

© 2023. Published by The Company of Biologists under the terms of the Creative Commons Attribution License (https://creativecommons.org/licenses/by/4.0/).

make this point more clear (lines 630ff). Please also refer to the GFP-expressing control sample shown in Figure 6A.

We counted a DiI injection site as showing floor-plate stalling when at least 50% of the fibers entering the floor plate failed to reach the exit site. Similarly, 'No turn' means that at least 50% of the axons at the exit site failed to turn rostrally. Because, these two phenotypes are not independent of each other (100% stalling prevents the analysis of the turning phenotype), we only did a statistical analysis for the DiI injection sites with correctly turning axons. We also would like to point out that we hardly had injection sites where it was difficult to decide whether the 50% threshold was reached or not.

-Fig. 2D. An scheme depicting the different phenotypes: "normal", "FP stalling" and "no turn" would help to understand the results. They can use schemes similar to those shown in Fig. 2K Parra et al. 2010.

We have added a scheme outlining the different phenotypes, as suggested to Figure 2A.

-Fig. 3A. The open-book drawing is confusing. It seems that they are analyzing open-book preparations in this experiment when this is not the case.

Now Figure 4: We have changed the schematic explaining our experimental design. We wanted to illustrate that we only took the dorsal-most part of the spinal cord, dissected from open-book preparations of the spinal cord, as explants to avoid the inclusion of other cell types.

-Fig. 3B. Authors claim that Cables1 is not required in pre-crossing axons as dsCables electroporation does not affect axonal growth of DiI neurons taken at HH22. However, to be sure that Cables1 mRNA levels are downregulated in pre-crossing axons, relative levels of Cables1 mRNA and/or protein should be also determined at HH22 not only at HH25.

We have clarified the quantification of downregulation efficiency. The qPCR data are taken from HH23, that is one day after electroporation. The Western blot data show differences in protein levels at HH25, that is 2 days after electroporation. In both cases, the downregulation efficiency is about 50%. This means that we got rid of all Cables1 mRNA, as we successfully transfected 50% of the cells in the targeted area (52.5% in n=4 embryos). The cell numbers were determined by counting the ratio of GFP-positive cells from transfected spinal cords in a single cell suspension.

-Fig. 4. The incapacity of Slit to induce axonal retraction in dsCables1 neurons is used to conclude that Cables1 is required to respond to Slit. However, downregulation of Cables1 by itself is even more effective inhibiting axonal growth than Slit treatment. Upon this strong effect as a background, it is difficult to assay slit response. Authors should point this observation in the manuscript.

We disagree. There is no significant difference between the neurite lengths between the control neurons in the presence of Slit and the neurons lacking Cables1 (dsCables1), p=023, or the neurons lacking Cables1 in the presence of Slit (dsCables1 and Slit), p>0.9999. As seen in the images and also from the measured neurite lengths, axons still show growth and further reduction would have been possible. We would also like to point out that the conclusion from this experiment is that Cables1 is required for the response of axons to either Slit or Wnt.

To support our claims, we have added another experiment addressing the need for Cables1 for post-crossing axons' responsiveness to Slit by downregulation of Robo receptors (Figure 10). These experiments confirmed that Slit/Robo signaling is required for the effect of Cables1 on post-crossing axons, in line with our final conclusion that Slit binding to Robo triggers internalization and Cables then transfers Abl from the C-term of Robo to β-Catenin. This results in phosphorylation of β-Catenin at tyrosine489 (β-Catenin pY489) and responsiveness to Wnt5a.

-Fig. 5B. In this Figure they do not differentiate between FP stalling or no turn phenotypes. A quantification taking into account the different phenotypes as shown in Fig.2D should be included.

© 2023. Published by The Company of Biologists under the terms of the Creative Commons Attribution License (https://creativecommons.org/licenses/by/4.0/).

Done, as suggested. This is Figure 6C in the revised manuscript.

-Fig. 6D,E. As postulated in the manuscript and based on the Rhee, et al. paper, the β-catenin phosphorylation is triggered by Abl quinase upon Slit-Robo signaling. How the authors explain then that isolated cells with axons growing on a plate recapitulate specific distal phosphorilation of β-catenin at Y489 in the absence of Slit signaling? This experiment shows that postcrossing axons contain more phosphorylated β-catenin as an intrinsic characteristic rather than as a consecuence of contact with floor plate signals. Authors should try a similar experiment but exposing the neurons (or explants) to Slit. Also, why β-catenin phosphorylation was not measured at the growth cone?

In Figure 6D and E (now Figure 7D,E), we compare pre- and post-crossing axons. Post-crossing axons do have 'a memory' of their contact with the floor plate, as this contact has changed the localization of Robo receptors to the surface (Philipp et al., 2012; Alther et al., 2016). Floor-plate contact also initiates differences in gene expression (e.g. Hhip expression in a Shh-and Glypican-dependent manner; Wilson and Stoeckli, 2013). The difference in Robo localization has also been described by others (Pignata et al., Cell Rep 29(2019)347).

In fact, the distal localization of pY-489 β-Catenin is in perfect agreement with our results: The localization of Robo1 on the distal portion of the axon is in line with published data from our own lab but also from the Castellani and the Tessier-Lavigne lab. Our results suggest that Cables is recruited to Abl bound to the C-term of Robo. Cables transfers Abl then to β-Catenin which is phosphorylated by Abl. Thus pY-489 β-Catenin would be localized predominantly where Robo is localized, i.e. the distal axon. In support of these results, experiments added to the revised version of the manuscript indicate that the response to Slit is required for the increase in β-Catenin pY489 (Figure 10B).

-Fig. 7. CAG::hrGFP electroporation is not specific for dl1 neurons. This experiment should be performed with Math1::tdTomatoF in order to analyze β-cat pY489 with or without dsCables1 specifically in dl1 neurons. Also, why GFP staining at the growth cones in Fig.7B is not visible in the axon?

As indicated in our schematic drawing (Figure 7A) we only cultured explants from the dorsal-most part of open-book preparations of spinal cords, making sure that our cultures are not mixtures with more ventral populations of neurons. We opted for CAG::hrGFP because Math1 is a weak promoter and the expression of GFP was very difficult to see after dissociating cells and culturing them in vitro. We used a GFP version that is not farnesylated to avoid interference with axonal staining of pY-489 β-Catenin. Therefore, GFP is not visible in axons with the imaging conditions used.

-Fig. 8. This experiment does not distinguish whether phosphorylated β-Cat is necessary for the correct navigation of post-crossing commissural axons (as it is claimed in the abstract) or it is also required for midline crossing. As it has been previously shown, correct navigation of post-crossing commisusal axons is a Wnt5 dependent process. As dsCables1 abrogates Wnt5a responsiveness (Fig.4B,C), does the phosphomimetic β-catenin Y489E construc rescue the Wnt5a response in dsCables1 electroporated neurons? Moreover, can the phosphomimetic β-catenin Y489E construc rescue the Slit response in dsCables1 electroporated neurons? Testing these effects on explants as in Fig. 4B,C but including phosphomimetic β-catenin, will help to understand to what extend phosphorylation of β-catenin is important for crossing, turning or both processes.

Yes, the phosphomimetic Y489E version of β-Catenin reduces the percentage of DiI injections sites with aberrant axonal navigation to control levels (Figure 9 in the revised manuscript). In contrast, a mutant version of β-Catenin that cannot be phosphorylated, β-CateninY489F, cannot rescue the axon guidance phenotype seen in the absence of Cables1.

-How do the authors envision the mechanism of Cables1/β-catenin mediated crossing and turning? A working model summarizing their hypothesis would help the reader to understand the results.

Minor points:
-Homogeneize the term "scale bars" or "bars" in the Figure Legends.

© 2023. Published by The Company of Biologists under the terms of the Creative Commons Attribution License (https://creativecommons.org/licenses/by/4.0/).

done

-Scale bar of insets in Fig.1C is missing.

The scale bar is now added, we apologize for the mistake.

-The antisense control for Cables probe should be shown at HH-22/24. Otherwise is not possible to distinguish whether they do not detect signal because is a negative control or because Cables1 is not expressed at HH25.

We have added the image of an adjacent section hybridized with the sense probe for HH25, in addition to HH22 to clarify that Cables expression is higher during floorplate crossing, exiting and turning rostrally but then levels decrease when post-crossing axons have initiated their growth along the rostro-caudal axis.

-Figure legend for Fig. 2D is missing

corrected

-Fig. 8B right panel is contaminated with growthing axons coming from the below DiI injection. Please replace the picture.

We have changed the outline of this figure.

-The quantification of the different phenotypes "FP stalling", "no turn" should be better explained in the Mat and Met section. The sentence " more than 50% of the axons...." is not clear. Was this measured by eye? Otherwise, please indicate the solware used to measure.

Yes, as mentioned above, it was hardly ever a close call. It is very easy for a person blind to the experimental condition to go through the DiI injection sites of an open-book preparation and to assess whether 50% or more of the axons that enter the floorplate reach the exit site, or not. Similarly, it is very easy to do the same for the turning behavior. We have changed the text describing this method of quantification to be more explicit (lines 630ff).

-Provide the quantification of the WB in Supplementary Fig. 2B normalising to Gapdh.

Added as Supplementary Figure 2C.

Reviewer #1 (Significance (Required)):
Previous results have demonstrated that Slit-induced modulation of adhesion is mediated by cables that links Robo-bound Abl kinase to N-cadherin-bound betacat (Rhee et al., 2007). Here the authors propose that a similar mechanism is operating in commissural neurons leave the midline after crossing and turn immediately after. The role of Cables in the process has not been previously addressed. Thus, after proper addressing of my main concerns, I consider this paper may advance in our knowlege of how growing axons navigate intermediate targets.

We appreciate this positive evaluation of our study and hope that the additional experiments and more detailed explanations have helped clarify open questions of the reviewer.

Reviewer #2 (Evidence, reproducibility and clarity (Required)):
In this paper entitled "Cables1 links Slit/Robo and Wnt/Frizzled signaling in commissural axon guidance", authors aim to the find the mechanisms the coordinate the floor plate exit and the rostral turning of commissural axons.

During development thousands of axons have to navigate long distances to reach their targets and build functional circuits. To facilitate their journey, their paths is divided into small portions by intermediated targets. The most studied intermediated target is the floorplate (FP) at the midline of the ventral spinal cord. Glia cells forming the FP express plethora of guidance cues. Commissural neurons, which have their cells bodies located in the dorsal part of the spinal cord, send their

© 2023. Published by The Company of Biologists under the terms of the Creative Commons Attribution License (https://creativecommons.org/licenses/by/4.0/).

axons towards the FP. These axons are first attracted by the FP which facilitate their entry within the FP. However, they switch this attractive response into a repulsive one in order to exit the FP and turn rostrally to connect their brain targets.

In order to ensure that this process will go smoothly, commissural axons have to adapt the composition of their receptors and the signaling pathways to switch from attractiveness to repulsion. So far, many processes have been involved such as the alternative splicing of receptors (Robo3; Chen et al Neuron 2008), protease regulation of receptor expression (Nawabi et al Genes & Dev 2010), trafficking of receptors, or their interaction profiles (Delloye et al Nat Neuro 2015). However, it is still not clear how 2 events (here exit from the FP and rostral turning) are linked.

Authors propose an original mechanism that involved the adaptor protein Cables 1. This protein has been shown to link the Robo/Slit1 signaling to Cadherins. Cables regulates the repulsive response to Slit and adhesion by the phosphoryla4on of b-Catenin by the kinase Abelson (Rhee et al Nat Cell Biol 2007).

The story developed here is very original and interesting: Cables would link the exit of FP (mediated by Robo/Slit signaling) and the rostral turning of the commissural axons (controlled by the Wnt/Fzd pathway. Below I'm proposing some experiments as many questions raised upon reading this beautiful work. The experiments are sound and could be reproducible. The statistic analysis looks fine.

We thank the reviewer for this positive assessment of our study.

I would suggest some experiments to strengthen the whole work:
•Authors might want to consider to perform some biochemistry experiments to show that Cables is able to interact with Robo1 and Fzd3: are these proteins in the same molecular complex? They could do 2 experiments: one in vitro by transfecting a cell line (such as HEK293 or cos cells) with plasmids coding for Robo1, Cables and Fzd3 or at least Cables and Fzd3 (as for Robo1/Cables they could refers to Rhee et al 2007). Another one would be in vivo: extracting proteins from the pre-crossing stage, the FP and post crossing stage; immunoprecipitation of Cables1 and see whether Robo1 and/or Fzd are pull down with Cables 1.

We decided not to do these experiments, as we felt that this would go beyond the current study. In fact, for our effects it is not necessary that Cables interacts physically with Robo or Fzd3. The important aspect is that Abl bound to Robo is transferred by Cables to β-Catenin. A direct interaction with Fzd3 is not necessary.

•From the pictures it seems that most of the axons are stalling in the FP when embryos are electroporated with dsCables1. It would be nice to show more examples of axons that are able to exit the FP but have turning problems. Given the data, as it is presented, it seems that Cables regulates more the FP exit (and therefore, as it was shown in Rhee et al, the responsiveness to Robo/Slit signaling).

The major phenotype is ‘no turn’. However, as we describe in response to reviewer 1 and in the manuscript, the ‘floorplate stalling’ and the ‘no turn’ phenotypes are not independent of each other. At DiI injection sites, where almost all axons stall in the floorplate, the turning cannot be assessed. Thus, the ‘no turn’ phenotype tends to be underestimated in conditions where floorplate crossing is also affected, as is the case after silencing Cables1.

In the same line, in Fig 4, Authors need to add a condition using dsCables and ds Fzd in order to see the effect of Cables on axon turning (response to Wnt). As it is this figure supports the role of Cables on FP exit but it's hard to make the link with commissural axon responsiveness to Wnt.

We belief that experiment 4 clearly demonstrates the absence of the Wnt responsiveness, as axons fail to grow in response to Wnt when they extend from neurons transfected with dsCables1 (Figure 4C). Because dsCables1 alone already abolishes all responsiveness to Wnt, the removal of Fzd at the same time would not change anything.

© 2023. Published by The Company of Biologists under the terms of the Creative Commons Attribution License (https://creativecommons.org/licenses/by/4.0/).

•Authors aim to show that Cables is a linker between 2 events: maybe it should be nice to try to disconnect these events. One way would be (if technically possible) to modulated expression of Cables at different stages. What would happen if Cables was down regulated upon FP crossing? Would axons still be able to respond to Wnt? The question I'm wondering about is whether the responsiveness to Slit and Wnt is acquired at the same time or whether axons should become sensitive to Slit and this event will prime them to respond to Slit. In order to address the following experiment could be performed: explants from HH22-HH23 embryos, could be treated with medium containing Slit first and then Wnt or vice et versa and perform some collapse assay.

Unfortunately, the experiment as proposed by the reviewer is not possible. The axons take on average 5.5 hours to cross the floorplate (entry – exit; Dumoulin et al., 2021). Most importantly, the protein that is already made before axons are at the exit site, could not be removed. Therefore, it is not possible to prevent the production of Cables only after axons have crossed the midline. As shown in Figure 1, Cables1 mRNA is present at HH22, that is when axons have reached and are about to enter the floorplate. We also do not belief that the in vitro experiment suggested by the reviewer would work. We would have to wash cell intensively to remove Slit added to the medium. This would interefere with their potential to grow in response to Wnt immediately after addition. However, we added experiments where we looked at the effect of Wnt after removal of Robo (Figure 10). These experiments demonstrate that responsiveness to Wnt can only be established when axons can respond to Slit, i.e. when Robo is activated.

•In Fig3 I was wondering whether post crossing axons were growing less because of the change in the regulation of adhesion: Rhee et al shows that Cables is able to modulate adhesion through N-cadherin. It would be interesting to perform immunostaining on these explant cultures to assess any change in adhesion molecules.

We have not found any changes in the expression levels of Contactin-2 (Axonin-1), NrCAM, or most importantly β1-Integrin, as our cultures grow on laminin.

•It is not clear whether Robo1 and/or Fzd induces the phosphorylation of b-catenin: is the Robo1/Slit binding induce the phosphorylation of b-cat and this event will prime the axons to respond to Wnt/Fzd? Or Wnt/Fzd is also able to control b-cat phosphorylation?

We have added an experiment, where we remove Robo1 from commissural neurons and compare pY489 β-Catenin levels (Figure 10). Furthermore, we demonstrate that in the absence of Robo1, Wnt has no stimulatory effect on axons (Figure 10C,D). These experiments supports our conclusion that Cables1 transfers Abl kinase from the C-terminal part of Robo to β-Catenin, which gets phosphorylated and thus is ready to act in the Wnt signaling pathway.

•The staining with the antibody needs to be detailed: as it is reported this antibody recognizes "a domain of Cables1 that is 90% identical to the corresponding region of Cables2": it seems that the Cables protein enrichment in the floor plate (around the central canal) is Cables 2 as its mRNA expression matches this profile of expression. The one expressed in the crossing axons might be Cables 1: one way to verify this, is to perform the staining on sections from embryos electroporated with dsCables 1. This is a very important control of the antibody to reinforce this point of the paper.

We belief that the staining of the cells around the central canal could be due to endfeet of precursors spanning the neural tube from the apical to the basal side. All cells seem to express some Cables1 (Figure 1B,C). As we did not find any effect of Cables2 on commissural axon navigation and we do not use antibodies to functionally interfere with Cables1 function, we did not do this experiment, as the antibody is not able to distinguish the two proteins. Most likely, there is little, if any, Cables2 expressed in the spinal cord during this time window. We still did some functional analyses but found no effect on axon guidance (Supplementary Figure 3).

•In Figures 3-4: why not performing some co culture of spinal cord explants with COS or HEK 293 cells expressing Slit1 or Wnt? This experiment will provide a clear-cut response to see the role of Cables in axon guidance. As there it is, Fig3 shows a role of Cables in axon growth but not guidance.

© 2023. Published by The Company of Biologists under the terms of the Creative Commons Attribution License (https://creativecommons.org/licenses/by/4.0/).

We respectfully disagree that in vitro experiment would help to show guidance versus growth. Guidance can only be shown in vivo. This is what we do. Our in vitro results are only included to address specific responsiveness of axons or expression changes in total β-Catenin or pY489 β-Catenin. But all our conclusions about the role of Cables in axon guidance are demonstrated in vivo. Experiments using co-cultures of axons with COS or HEK cells would be impossible to control for timing and amount of Slit or Wnt release.

•In Figure 6: my understanding of axon guidance is that every guidance decision happens at the level of the growth cone. However, it seems that in post crossing stage, there is a strong decrease of b-cat and phosphor- b cat within the growth cone compared to the precrossing stage. If beta cat is the effector of Cables to link Robo/Slit and Wnt/Fzd signaling I would expect it to be localized at the growth cone. I think authors should discuss this point. Regarding the normalization, it would be better to counterstaing the neurons with actin and use the measure of its fluorescence to normalize phopho-beta cat.

There must be a misunderstanding. We do not demonstrate or claim that there is a decrease in β-Catenin or pY489 β-Catenin between pre- and post-crossing axons. We only demonstrate that the distribution of pY489 β-Catenin is clustered in distal post- but not pre-crossing axons. This change in localization of pY489 β-Catenin is supporting our model that Cables1 transfers Abl kinase to β-Catenin and phosphorylates it and prepare it for signaling in the Wnt pathway. And, as demonstrated pY489 β-Catenin and β-Catenin are in the growth cone. However, for quantification we concentrated on the axon, as the difference in growth cone morphology would have complicated the quantification.

Minor points:
•In figure 2: it seems that there are few axons labelled with DiI in the dsCables1 condition (Fig2B): it would be the choice of the picture or maybe the downregulation of Cables 1 interfere with the survival of dl1 neurons (even though in supp 1C it is shown that most of the populations are still there with no difference with the control side) or maybe some axons are delayed to reach to FP on time: the picture is focused on the FP: are there any axons still growing in the side of the open book preparation? Again, the picture that could be misleading.

We have exchanged the images for alternatives with a better matched number of DiI-labelled axons. There is indeed no evidence for cell death, as axons are still there at normal numbers when we analyze open-book preparations a day later than usually. The difference in the number of axons labelled by DiI is only due to the variability in the amount of DiI injected per injection site.

•In Fig1 legends, maybe Authors wanted to write "At HH18 dl1 commissural neurons start to extend their axons in the ventral spinal cord"?

No, what we mean is, as shown in Figure 1A, that axons emerge from the cell body at this time. They reach the ventral spinal cord by HH21 and the floor plate by HH22.

•I would also remove the yellow shadow on the Fig1A: it could be misleading as at first glance the reader might wonder whether there are 2 populations of dl1 neurons.

We have done as suggested to make the image clearer.

Reviewer #2 (Significance (Required)):
It is still not clear how axons cross the midline. So far, many processes have been involved such as the alternative splicing of receptors (Robo3; Chen et al Neuron 2008), protease regulation of receptor expression (Nawabi et al Genes & Dev 2010), trafficking of receptors, or their interaction profiles (Delloye et al Nat Neuro 2015). However, it is still not clear how 2 events (here exit from the FP and rostral turning) are linked.
Authors propose an original mechanism that involved the adaptor protein Cables 1. This protein has been shown to link the Robo/Slit1 signaling to Cadherins. Cables regulates the repulsive response to Slit and adhesion by the phosphorylation of b-Catenin by the kinase Abelson (Rhee et al Nat Cell Biol 2007).

© 2023. Published by The Company of Biologists under the terms of the Creative Commons Attribution License (https://creativecommons.org/licenses/by/4.0/).

The audience that will be interested in this work is the neurodevelopment filed, axon regeneration field and overall people interested in neuronal circuit formation and function.
My field of expertise is molecular and cellular neuroscience applied to axon guidance (crossing the FP) in mice models, axon regeneration and circuit formation.

We are happy to learn about the positive assessment of our work by a specialist.

Reviewer #3 (Evidence, reproducibility and clarity (Required)):
In their manuscript, Zuniga and Stoeckli characterize the role of Cables in commissural axon guidance in the developing chick spinal cord. Based on a combination of in vitro outgrowth assays and in vivo dye-tracing experiments, the authors propose that Cables participates in both normal repulsive responses to Slit and attractive responses to Wnt5. Using combinations of low-does knock down of cables/robo and or B-catenin, the author suggest an in vivo link between these pathways. Using IF with phospho-specific antibodies to B-catenin, the authors suggest that there is elevated P-Bcatenin in the post-crossing segments of distal axons. While potentially interesting, the present study falls short of adequately supporting the major claims. In addition, there are several instances where experiments lack appropriate controls.

Specific Comments

The conclusions reached by the authors are over-stated given the experiments performed. For example, the authors describe 'silencing' cables throughout the paper; however, the knock down that they achieve is approximately 50%. Indeed, it is quite surprising that such strong effects on growth/guidance can be achieved with a two-fold depletion of the gene product. Nevertheless, the rescue experiments provide nice evidence that dsRNA for Cables is causing a phenotype. This partial knockdown precludes strong conclusions, like for Figure 3, where they state that 'Cables is not required for pre-crossing.' The language needs to be tempered.

We rephrased the paragraph where we describe the effect of Cables 1 and the efficiency of downregulation to stress that the parameters that we use for electroporation result in around 50% of the cells successfully transfected (lines 154 – 162, and legend of Supplementary Figure 2). Therefore, to find mRNA levels and protein levels reduced to about half indicates that our method is extremely efficient and removes the targeted mRNA and the protein almost completely. We need to point out here that we always analyze the temporal expression pattern to electroporated embryos before the protein of interest has accumulated, as in ovo RNAi obviously does not remove protein but only prevents translation and therefore the synthesis of new protein. As proteins can be extremely stable compared to the time line of embryonic development, we inject and electroporate dsRNA before we find expression of mRNA.

Figure 4: the authors use bath application of Slit and Wnt to test effects of cables on Slit and Wnt responses. The observed effect sizes are very small and a single assay of this type does not allow such strong conclusions like 'loss of Cables prevents responsiveness.' Again, it is difficult to imagine that 50% reduction would completely prevent responses, raising questions about the suitability of this assay for measuring responsiveness- perhaps growth cone collapse would give more convincing results.

As mentioned above, we are almost completely eliminating the targeted protein in the transfected neurons. For the explants, we only looked at the neurons expressing td-Tomato driven by the Math1 promoter. Thus, these neurons were transfected. Obviously, we cannot be sure that 100% of our cells took up the plasmid and the dsRNA, but the chances are very high that this is the case based on the ration between plasmid and dsRNA.

Figure 5: The authors should more clearly document the effects they are seeing in these manipulations. As written, all we know is that there are 'significant effects on axon guidance.' What are these effects? Do they see the predicted differences between robo/cables and Bcatenin/cables phenotypes? e.g re-crossing defects in the case of robo and anterior turning defects in the case of B-catenin?

© 2023. Published by The Company of Biologists under the terms of the Creative Commons Attribution License (https://creativecommons.org/licenses/by/4.0/).

We have added the analysis of the detailed axon guidance problems seen in the absence of Robo1, Cables1, βCatenin, or combinations, now Figure 6C. Indeed, we find that the phenotype 'no turn' is more prevalent in the condition with loss of both Cables and βCatenin. However, as mentioned above in response to a question raised by Reviewer 2, the two phenotypes are not independent of each other. Stalling in the floor plate of the majority of axons prevents the analysis of the turning phenotype. That is why we only use the 'normal' DiI injection sites for the statistical analysis.

Also related to Figure 5:
The authors do not validate the dsRNA knockdown of either Robo or B catenin.
It is unclear what the interpretation or expectation of the triple knock down condition is.

We have used the same ESTs to produce dsRNA derived from Robo and βCatenin in our previous publications (Alther et al., Development 143(2016)994; Avilés and Stoeckli Dev Neurobiol 76(2016)190). Therefore, we only repeated the functional experiments to verify reproducibility of the effect but we did not quantify the efficiency of downregulation in detail again.

Figure 6: For this reviewer images showing enhanced P-Catenin in post-crossing distal axons is not convincing. The differences are not obvious by eye and the quantification suggests an ~30% increase. In contrast a nearly 4-fold increase is reported in Figure 7 for this same measurement. This raises concerns about the reproducibility of this 'phenotype.

Staining intensities are subject to batch-to-batch variability. Therefore, the experiments shown in Figure 7 (Figure 6 in the original manuscript) cannot be directly compared to the levels in Figure 8 (previously Figure 7). However, within the experiments, we carefully normalized data. We do not make any claims about absolute staining intensities.

Also related to Figure 6:
No validation of antibody specificity is provided or described.

Again, please keep in mind that we do not make any claims about absolute values. All are results are based on stainings with the same antibody and comparison between different areas of the same axons. Therefore, the specificity of the antibody is important but not a fundamental aspect of our results.

Figure 8: As for figure 5, phenotypic documentation is incomplete. In addition, no controls are shown to assure that the different mutant forms of B-catenin are comparably expressed, nor is there an unmutated wild-type control. The authors state that expression of these constructs alone has no effect on normal guidance; however, the supplemental data 6B would seem to indicate that both forms lead to increases abnormal phenotypes.

There is an increase in the number of injection sites with aberrant axon guidance, however, this was not significant. We cannot exclude the possibility that premature expression, or overexpression of βCatenin pY489E or βCatenin pY489F does interfere with the endogenous βCatenin pY489. We still decided to keep these experiments in the revised version of the manuscript as they support our conclusion that Cables1 is required for axonal responsiveness to Slit and Wnts, and that this effect is mediated by phosphorylation of βCatenin at Y489. We are aware that this experiment in isolation is not sufficient.

Reviewer #3 (Significance (Required)):
The work builds on in vitro observa4ons from Rhee, 2007 about links between Robo signaling and Cables func4on. If adequately demonstrated, integra4on and coordina4on of Robo and Wnt axon guidance pathways is quite significant.

We thank the reviewer for this positive assessment.

© 2023. Published by The Company of Biologists under the terms of the Creative Commons Attribution License (https://creativecommons.org/licenses/by/4.0/).

Original submission

First decision letter

MS ID#: DEVELOP/2023/201671

MS TITLE: Cables1 links Slit/Robo and Wnt/Frizzled signaling in commissural axon guidance

AUTHORS: Esther Stoeckli, Nikole Zuniga, Alexandre Dumoulin, and Giuseppe Vaccaro

I asked the original referees from Review Commons to assess your revisions to this study. I have now received their reports and I have reached a decision. The referees' comments are appended below, or you can access them online: please go to BenchPress and click on the 'Manuscripts with Decisions' queue in the Author Area.

As you will see, the referees express considerable interest in your work, but continue to have some significant criticisms and recommend a substantial revision of your manuscript before we can consider publication. The referees are convinced of the evidence for a role for Cables in commissural axon guidance but they are less convinced by the proposed connection between Slit-Robo and Wnt-b-catenin. I suggest revising the phrasing of your manuscript to avoid overstatement of the findings and conducting experiments that strengthen this conclusion to address the criticisms raised by Referees 1 and 3.

If you are able to revise the manuscript along the lines suggested, which may involve further experiments, I will be happy receive a revised version of the manuscript. Your revised paper will be re-reviewed by one or more of the original referees, and acceptance of your manuscript will depend on your addressing satisfactorily the reviewers' major concerns. Please also note that Development will normally permit only one round of major revision. If it would be helpful, you are welcome to contact us to discuss your revision in greater detail. Please send us a point-by-point response indicating your plans for addressing the referee’s comments, and we will look over this and provide further guidance.

Please attend to all of the reviewers' comments and ensure that you clearly highlight all changes made in the revised manuscript. Please avoid using 'Tracked changes' in Word files as these are lost in PDF conversion. I should be grateful if you would also provide a point-by-point response detailing how you have dealt with the points raised by the reviewers in the 'Response to Reviewers' box. If you do not agree with any of their criticisms or suggestions please explain clearly why this is so.

Reviewer 1

Advance summary and potential significance to field

In this work, authors aim to address the mechanisms coordinating the floor plate exit and the rostral turning of commissural axons in the developing spinal cord. Authors propose an original mechanism: the adaptor protein Cables 1 links the Robo/Slit1 (signal to exit the floor plate) signaling to Wnt (signal to turn rostrally).
Cables regulates the repulsive response to Slit and adhesion by the phosphorylation of b-Catenin by the kinase Abelson (Rhee et al Nat Cell Biol 2007).

Comments for the author

Even if the title and the suggested idea are very appealing, no direct evidence backs up these claims. This is my main concern. There are only correlations. Therefore, Authors should be careful with the phrasing and they should not overstate their findings. Either they change the phrasing of the whole text (and the related title) or they could perform the suggested experiments.
I would suggest some experiments to strengthen the whole work:

© 2023. Published by The Company of Biologists under the terms of the Creative Commons Attribution License (https://creativecommons.org/licenses/by/4.0/).

- From the pictures it seems that most of the axons are stalling in the FP or cannot exit the FP when embryos are electroporated with dsCables1. Therefore, it's confusing to state the Cables1 controls the turning response to Wnt: if axons cannot exit the floorplate, then the next step is expected to be compromised. In Lyuksyutova et al 2003, the turning phenotype is very clear upon Wnt signaling modulation. However, these phenotypes are not observed in the present work (even at later stages of development). Cables might be involved in Slit and Wnt responses independently but is really Cables their linker? It's hard to say regarding the given data.
- Cables1 is enriched in the dl1 population. As authors are able to express fluorescent reporter in this specific population, they could address b-catenin phosphorylation in dl1 neurons specifically (sup fig 6).

Moreover, it is not clear why in control condition (total b-catenin- sup FigE- neurons are stained with anti-neurofilmant and tuj1 is used to analyze b-catenin phosphorylation on pY489 (sup Fig 6F). This image is also confusing as tuj1 staining matches pY489. One would expect tuj1 expression to be homogenous all along the neurite. Are there any chances of cross reaction between antibodies? Authors state the b-catenin expression is homogenous along the axon: from Sup Fig 6E, b-catenin is enriched in the growth cone but is barely expressed along the axons.

- In Figures 7: Guidance decision happens at the level of the growth cone. However, it seems that in post crossing stage, there is a strong decrease of b-catenin and phospho-b catenin within the growth cone compared to the pre-crossing stage. If beta catenin is the effector of Cables to link Robo/Slit and Wnt/Fzd signaling I would expect it to be localized at the growth cone.
- Figure 8: In 8B, there is not much differences in the distribution of phospho-b-catenin. I would suggest to counterstain with phalloidin (actin). Moreover, regarding the distribution of phospho-b-catenin expression it seems that several populations of commissural neurons have been analyzed. I would suggest to focus only on dl1 neurons. Regarding the normalization, it would be better to counterstain neurons with actin and use it to normalize phospho-beta cat fluorescence. It would be also important to compare proximal control vs Proximal ds-cables1 and distal control vs distal ds-cables1

Minor points:

- Please use "electroporated" cells instead of "transfection" as it implies that the DNA enter the cells with a chemical (transfection agent). Lines 162-173 for example.
- Figure 4 could be combined with figure 5.
- Figure 9 could be enriched with some illustrations.

Reviewer 2

Advance summary and potential significance to field

This work by Zuñiga et al. demonstrate that commissural axons need Cables1 to leave the floor plate and turn ascending to the brain. Cables1 acts as a linker of two key axon guidance pathways (Slit and Wnt) during this process. Cables1 would localize phosphorylated β-catenin to the distal axon being this necessary for the correct turning of postcrossing commissural axons. This is an step forward to understand how axons navigate during development and in particular how commissural spinal neurons turn after midline crossing.

Comments for the author

I think the authors have answered all the requirements and concerns. I congratulate them for the effort and for the nice work.

Reviewer 3

Advance summary and potential significance to field

The authors provide a convincing demonstration of the role of Cables in commissural axon guidance in the developing spinal cord. Further the authors identify phosphorylation of the B-catenin as an

© 2023. Published by The Company of Biologists under the terms of the Creative Commons Attribution License (https://creativecommons.org/licenses/by/4.0/).

important output of Cables that may explain how it contributes to Wnt-Frizzled signaling in this context.

Comments for the author

In their manuscript, Zuniga et al., characterize the role of Cables in commissural axon guidance in the developing chick spinal cord. Based on a combination of in vitro outgrowth assays and in vivo dye-tracing experiments, the authors propose that Cables participates in both normal repulsive responses to Slit and attractive responses to Wnt5. Using combinations of low-does knock down of cables/robo and or B-catenin, the authors suggest an in vivo link between these pathways. Using IF with phospho-specific antibodies to B-catenin, the authors show that there is elevated P-Bcatenin in the post-crossing segments of distal axons. The findings documenting a role for Cables in commissural axon guidance are clear and compelling and include nice live-imaging experiments. In addition, the authors do a nice job of controlling for other potential effects of Cable knock-down that could confound interpretation of the guidance phenotypes. However, the proposed in vivo links to Slit-Robo and Wnt-B-catenin are less clear given the described phenotypes. In addition, there are several instances where experiments lack appropriate controls. If the authors can address some of the comments below, I believe the manuscript could be a much stronger candidate for publication.

Major Comments
The primary observations implicating Cables in both Slit and Robo responses are in vitro. The in vivo data is confusing on this point. For example, loss of Robo, predominantly leads to FP stallin (based on work in mice), while Wnt-Frizzled disruoption leads to randomization of turning in post-crossing axons. Here in the genetic interaction experiments the predominant phenotype observed is a failure to turn (both for Robo and Wnt/Bcat). This is confusing- shouldn't the phenotype reflect distinct stages of defects based on the proposed model?
In addition, the way the phenotypes are quantified makes it very difficult to make the appropriate comparisons- % of injection sites with phenotypes does not allow the reader to distinguish between the expected differences in the types of defects that are observed. Comparisons of % of axons exhibiting one behavior or the other would be helpful.

Specific comments:
Cables knockdown and expression pattern: the authors conclude that the two fold-reduction in protein that they see by Western coupled with the % of transfected cells means that Cables is completely gone from transfected cells. This is not particularly convincing. Perhaps an IF for Cables in SC's with knockdown would be more convincing AND would serve the dual purpose of confirming Cables Ab specificity on spinal cord sections.
In Figure 2, the authors rescue the Cables knock-down by over-expressing a RNAi resistant form of Cables. What is the effect of over-expressing Cables alone in an otherwise wild-type background?
Figure 5: The authors use bath application of Slit and Wnt to test effects of cables on Slit and Wnt responses. The observed effect sizes are very small and a single assay of this type does not allow such strong conclusions like 'loss of Cables prevents responsiveness.' Cables knockdown appears to lead to a strong reduction in axon growth (the effect is greater than the effect of Slit treatment). Could there be a ceiling/floor effect on the outgrowth response, such that the extent of outgrowth in cables ko can not be further enhanced by Slit addition? Perhaps growth cone collapse for Slit and growth cone turning for Wnt would give more convincing results.
Figure 6: The authors should more clearly document the effects they are seeing in these manipulations. As written, all we know is that there are 'significant effects on axon guidance.' What are these effects? Do they see the predicted differences between robo/cables and B-catenin/cables phenotypes? e.g stalling defects in the case of robo and randomization of post-crossing turning in the case of B-catenin? (See also major comment above)

Also related to Figure 6:
The authors do not validate the dsRNA knockdown of either Robo or B catenin.
It is unclear what the interpretation or expectation of the triple knock down condition is.
Figure 7 and 8: The differences of phospho-catenin between proximal and distal in HH26 quantification suggests an ~30% increase. In contrast a nearly 4-fold increase is reported in Figure 8 for this same measurement. This raises concerns about the reproducibility of this pattern. Some

© 2023. Published by The Company of Biologists under the terms of the Creative Commons Attribution License (https://creativecommons.org/licenses/by/4.0/).

comment on why this measurement is so different in the two 'control' conditions would be helpful. This is especially true given the very small effect of Robo Ko on P-Catenin levels shown in Figure 10

Also related to Figure 7 and 8:
Perhaps I missed it, but no validation of antibody specificity is provided or described.
Figure 9: As for figure 5, phenotypic documentation is incomplete. In addition, no controls are shown to assure that the different mutant forms of B-catenin are comparably expressed, nor is there an unmutated wild-type control. It would be helpful to indicate on the histogram which comparisons lead to significant differences given the huge scatter of the data points.
Finally, if as the authors suggest phosphorylation of B-catenin is what drives the switch from Slit response to Wnt response, why doesn't over-expression of the phosphomimetic B-catenin lead to aberrant premature anterior turning on the ipsilateral side of the spinal cord? Perhaps visualizing the distribution of the over-expressed B-cat variants as suggested above might offer some insight into this curiosity.

First revision

Author response to reviewers' comments

Point-to-point response to reviewers' comments

We appreciate constructive criticism by the reviewers to our manuscript that was originally submitted to Reviewer Commons and then in revised form to Development. The current version is a revised version of the manuscript sent to Development.

Reviewer 1 Comments for the Author:

Even if the title and the suggested idea are very appealing, no direct evidence backs up these claims. This is my main concern. There are only correlations. Therefore, Authors should be careful with the phrasing and they should not overstate their findings. Either they change the phrasing of the whole text (and the related title) or they could perform the suggested experiments.

I would suggest some experiments to strengthen the whole work:

•From the pictures it seems that most of the axons are stalling in the FP or cannot exit the FP when embryos are electroporated with dsCables1. Therefore, it's confusing to state the Cables1 controls the turning response to Wnt: if axons cannot exit the floorplate, then the next step is expected to be compromised. In Lyuksyutova et al 2003, the turning phenotype is very clear upon Wnt signaling modulation. However, these phenotypes are not observed in the present work (even at later stages of development). Cables might be involved in Slit and Wnt responses independently but is really Cables their linker? It's hard to say regarding the given data.

The reviewer is correct in pointing out that the turning response is affected, when axon cannot exit the floor plate. We do not quite understand what the reviewer is referring to when saying that the 'turning phenotype is very clear upon Wnt signaling modulation. However, these phenotypes are not observed in [our work].' We do see a 'no turn' phenotype at about 50% of the DiI injection sites after silencing Cables1. This is exactly reproducing the 'knotting/stalling' phenotype described in the absence of Wnt signaling by Lyuskyutova et al., 2003. Furthermore, the phenotype that we found after knocking down Cables1 was reproducing the phenotype that we found after interference with Wnt signaling in our previous studies in chicken embryos (Domanitskaya et al., 2010, J. Neurosci. 30, 11167-11176, and Avilés and Stoeckli, 2016, Dev. Neurobiol. 76, 190-208).

In line with the expectation of a role of Cables1 in Robo-mediated signaling, we found an increase in the percentage of DiI injection sites where axons failed to reach the floorplate exit site. We are not sure that we understand what the reviewer means by saying that he/she beliefs that Cables is

© 2023. Published by The Company of Biologists under the terms of the Creative Commons Attribution License (https://creativecommons.org/licenses/by/4.0/).

involved in Slit and Wnt signaling but doubts that 'Cables is their linker'. Of course, only looking at Figure 2 does not allow for the conclusion that Cables1 connects the two signaling pathways. However, our hypothesis is supported by the sum of the experiments, in particular the demonstration that we need phosphorylation of β-Catenin at tyrosine 489 (β-Catenin pY489) and its localization in the distal axon/growth cone for rostral turn of post-crossing commissural axons. This phosphorylation depends on Cables1 (Figure 8). Furthermore, knockdown of Robo1 also prevents the accumulation of β-Catenin pY489 and the responsiveness to Wnt (Figure 10) making Cables1 the connection between the two signaling pathways. We have summarized this hypothesis in a schematic drawing added as Figure 11.

•Cables1 is enriched in the dl1 population. As authors are able to express fluorescent reporter in this specific population, they could address b-catenin phosphorylation in dl1 neurons specifically (sup fig 6). Moreover, it is not clear why in control condition (total b-catenin- sup FigE- neurons are stained with antineurofilmant and tuj1 is used to analyze b-catenin phosphorylation on pY489 (sup Fig 6F). This image is also confusing as tuj1 staining matches pY489. One would expect tuj1 expression to be homogenous all along the neurite. Are there any chances of cross reaction between antibodies? Authors state the b- catenin expression is homogenous along the axon: from Sup Fig 6E, b-catenin is enriched in the growth cone but is barely expressed along the axons.

In Supplementary Figure 6, we analyzed the distribution in wild-type neurons without any experimental manipulation. Thus, we did not want to express Math1::GFP. However, we are confident that the neurons shown in Supplementary Figure 6 are dI1 neurons based on our protocol used to dissect them from open-book preparations of spinal cords. In response to the comment of the reviewer, we provide a new version of Supplementary Figure 6, where we confirm the identity of the neurons as dI1 neurons by using Robo3 staining, a marker for dorsal commissural neurons, instead of neurofilament or βIII-Tubulin staining. We show two neurons for each staining group: β-Catenin and β-Catenin pY489. In both cases, the identity of the neurons as commissural neurons in confirmed by Robo3 staining. As described in our manuscript, the levels of β-Catenin are homogenous along the entire axon, whereas β-Catenin pY489 is found at higher levels in the distal axon. These results are consistent with those shown in Figures 7, 8 and 10.

•In Figures 7: Guidance decision happens at the level of the growth cone. However, it seems that in post crossing stage, there is a strong decrease of b-catenin and phospho-b catenin within the growth cone compared to the pre-crossing stage. If beta catenin is the effector of Cables to link Robo/Slit and Wnt/Fzd signaling I would expect it to be localized at the growth cone.

We do not understand what the reviewer refers to here. There is no decrease in either β-Catenin or β- Catenin pY489 in post-crossing axons. There is more β-Catenin pY489 in growth cones of post-crossing axons. However, because growth cone size would make a comparative measurement difficult, we decided to compare levels of distal (normalized to proximal) β-Catenin and β-Catenin pY489, respectively. In response to the reviewer's comment, we have added Supplementary Figure S8, where we demonstrate the localization of β-Catenin pY489 in the growth cone in comparison to Contactin2/Axonin-1.

•Figure 8: In 8B, there is not much differences in the distribution of phospho-b-catenin. I would suggest to counterstain with phalloidin (actin). Moreover, regarding the distribution of phospho-b-catenin expression, it seems that several populations of commissural neurons have been analyzed. I would suggest to focus only on dl1 neurons. Regarding the normalization, it would be better to counterstain neurons with actin and use it to normalize phospho-beta cat fluorescence. It would be also important to compare proximal control vs Proximal ds-cables1 and distal control vs distal ds-cables1

We decided to not counterstain the axons for the measurements of β-Catenin pY489 axons, as we wanted to prevent strong phalloidin staining to obscure differences in β-Catenin pY489 levels. We can assure the reviewer that we took great care in only dissecting the dorsal dI1 commissural neurons (as indicated in Figure 8A with the blue circles). This can also be verified by looking at the staining provided in Supplementary Figures 6 and 8, where Robo3 and Contactin2, respectively, was used to verify dI1 identity. We did compare proximal control vs proximal dsCables1 levels. There is no significant difference (p=0.17). In contrast, there is a significant difference between

© 2023. Published by The Company of Biologists under the terms of the Creative Commons Attribution License (https://creativecommons.org/licenses/by/4.0/).

distal control vs distal dsCables levels: p<0.0001, in agreement with our model that Cables is required for the phosphorylation and localization of β-Catenin pY489 in distal axons.

Minor points:

•Please use “electroporated” cells instead of “transfection” as it implies that the DNA enter the cells with a chemical (transfection agent). Lines 162-173 for example.

done

•Figure 4 could be combined with figure 5.

We disagree. Figures 4 and 5 are not addressing the same issue. We find it easier to keep the absence of an effect on growth of pre-crossing axons and the effect of Cables1 on Wnt and Slit responsiveness separate.

•Figure 9 could be enriched with some illustrations.

We added a summary of our model as Figure 11, instead of adding it to Figure 9.

Reviewer 2 Advance Summary and Potential Significance to Field:

This work by Zuñiga et al. demonstrate that commissural axons need Cables1 to leave the floor plate and turn ascending to the brain. Cables1 acts as a linker of two key axon guidance pathways (Slit and Wnt) during this process. Cables1 would localize phosphorylated β-catenin to the distal axon being this necessary for the correct turning of postcrossing commissural axons. This is an step forward to understand how axons navigate during development and in particular how commissural spinal neurons turn after midline crossing.

Reviewer 2 Comments for the Author:

I think the authors have answered all the requirements and concerns. I congratulate them for the effort and for the nice work.

We thank the reviewer for this positive assessment of our studies.

Reviewer 3 Comments for the Author:

In their manuscript, Zuniga et al., characterize the role of Cables in commissural axon guidance in the developing chick spinal cord. Based on a combination of in vitro outgrowth assays and in vivo dye- tracing experiments, the authors propose that Cables participates in both normal repulsive responses to Slit and attractive responses to Wnt5. Using combinations of low-does knock down of cables/robo and or B-catenin, the authors suggest an in vivo link between these pathways. Using IF with phospho-specific antibodies to B-catenin, the authors show that there is elevated P-Bcatenin in the post-crossing segments of distal axons. The findings documenting a role for Cables in commissural axon guidance are clear and compelling and include nice live-imaging experiments. In addition, the authors do a nice job of controlling for other potential effects of Cable knock-down that could confound interpretation of the guidance phenotypes. However, the proposed in vivo links to Slit-Robo and Wnt-B-catenin are less clear given the described phenotypes. In addition, there are several instances where experiments lack appropriate controls. If the authors can address some of the comments below, I believe the manuscript could be a much stronger candidate for publication.

Major Comments

The primary observations implicating Cables in both Slit and Robo responses are in vitro. The in vivo data is confusing on this point. For example, loss of Robo, predominantly leads to FP stallin (based on work in mice), while Wnt-Frizzled disruoption leads to randomization of turning in post-

© 2023. Published by The Company of Biologists under the terms of the Creative Commons Attribution License (https://creativecommons.org/licenses/by/4.0/).

crossing axons. Here in the genetic interaction experiments the predominant phenotype observed is a failure to turn (both for Robo and Wnt/Bcat). This is confusing- shouldn't the phenotype reflect distinct stages of defects based on the proposed model?

As the reviewer points out, we use both in vitro and in vivo experiments to demonstrate an involvement of Cables in both Robo/Slit and Wnt signaling. Whereas the in vitro experiments allow us to separate effects of Slit and Robo on post-crossing commissural axons, we also provide in vivo evidence. For example, Figure 10 demonstrates that without Robo signaling, Cables-mediated accumulation of β- Catenin pY489 does not happen.

With respect to the in vivo phenotype, we would like to point out that the two phenotypes 'stalling in the floorplate' and 'no turn' are not independent of each other. A DiI injection site, where almost all axons stall in the floorplate, cannot be assessed with respect to the turning phenotype, as too few axons would reach the contralateral floorplate border. This is one of the reasons for addressing the responsiveness to Robo and Wnt of axons with and without Cables1 in vitro. There the responsiveness can be assessed separately. However, we believe that our results are in very good agreement with the expectation that loss of Robo function results in stalling, whereas loss of Wnt signaling predominantely (but not exclusively, see our previous results Domanitskaya et al., J Neurosci 30(2010)11167-11176; Avilés and Stoeckli, Dev Neurobiol 76(2016)190-208) affects turning. A higher percentage of DiI injection sites with axons stalling in the floorplate after downregulation of Robo1 would have been possible by using younger embryos at the time of electroporation (see Alther et al., Development 143(2016) 994- 1004). However, for the demonstration of collaboration between Cables1 and Robo1 or Cables1 and Wnt signaling, we wanted to use always the same protocol and we did not want the effect of Robo1 to prevent any later analysis of post-crossing axon trajectories. With the parameters used in this study, we could analyze both midline crossing and turning into the antero-posterior axis.

Finally, we would also like to mention that knotting/stalling was observed very frequently in the absence of Wnt signaling in the mouse (fzd3 knockout) by Lyuksyutova et al., 2003. The fact that random turning is rare in chicken embryos is most likely explained by the strong effect of Shh. Shh forms a rostral^{low} to caudal^{high} gradient and repels post-crossing commissural axons (Bourikas et al., Natue Neurosci. 8(2005)297-304). This effect is strongest at the lumbar level of the spinal cord, where we do our analysis (Domanitskaya et al., J. Neurosci. 30(2010)11167-11176). In mice, more rostral levels are usually used and the Shh effect may be relatively weaker compared to the Wnt effect. In chicken embryos, randomization of turns was seen in the absence of Shh (Bourikas et al., 2005).

In addition, the way the phenotypes are quantified makes it very difficult to make the appropriate comparisons- % of injection sites with phenotypes does not allow the reader to distinguish between the expected differences in the types of defects that are observed. Comparisons of % of axons exhibiting one behavior or the other would be helpful.

As mentioned above, the two phenotypes 'stalling in the floor plate' and 'no turn' are not independent of each other. An axon that fails to exit the floor plate cannot be assessed for its ability to turn along the Wnt gradient along the contralateral floor-plate border. Thus, we cannot count axons and compare the number of axons exhibiting one versus the other behavior. We belief that our way of quantifying the phenotypes is the best option. Every DiI injection site is a random selection of dI1 axons. Based on our electroporation efficiency, about 50% of those axons will lack the targeted gene product. Therefore, we carry out the quantification as described in detail in the Method section. When 50% of the axons of a DiI injection site stall in the floorplate, we count this injection site as 'stalling in the floorplate'. If the majority of the axons reaching the floorplate exit site fail to turn rostrally, we count this DiI injection site as 'no turn'. If by chance 95% or more of the axons labelled at a given DiI injection site are stalling in the floorplate, we cannot assess their potential to turn at the exit site. Therefore, we use only the DiI injection sites with normal trajectories for our quantification with statistical analyses. The 'qualitative' phenotypes are shown in Figure 2D, Figure 6C, Supp. Figure 5B, and Figure 9B, respectively.

Specific comments:

Cables knockdown and expression pattern: the authors conclude that the two fold-reduction in

© 2023. Published by The Company of Biologists under the terms of the Creative Commons Attribution License (https://creativecommons.org/licenses/by/4.0/).

protein that they see by Western coupled with the % of transfected cells means that Cables is completely gone from transfected cells. This is not particularly convincing. Perhaps an IF for Cables in SC's with knockdown would be more convincing AND would serve the dual purpose of confirming Cables Ab specificity on spinal cord sections.

Unfortunately, the quality of the antibody staining is not sufficiently quantitative to demonstrate a reduction of the staining to 50%. Again, keep in mind that we expect a 'checker board' pattern, as some cells will maintain the protein level, whereas neighbors will lack staining. We would need single cell resolution to show that some cells have lost Cables1 completely whereas other cells still produce normal levels. Therefore, we chose to quantify the protein levels by Western blot and mRNA levels by qRT-PCR. We detail our calculations in the legend of Supplementary Figure S2. We counted the number of successfully targeted cells in two batches of electroporated embryos (n=4 in each batch). The measured levels of Cables mRNA and protein both are in line with our conclusion that targeted cells (~50%, counted in two batches of 4 embryos each) lose Cables1 expression.

In Figure 2, the authors rescue the Cables knock-down by over-expressing a RNAi resistant form of Cables. What is the effect of over-expressing Cables alone in an otherwise wild-type background?

Overexpression of mouse Cables1 in dI1 neurons (Math1::mCables1) does not affect axon guidance. We have done these experiments as suggested by the reviewer and added the results to Figure 2. Normal dI1 trajectories are found at 75% of the DiI injection sites after expression of mouse Cables1 in a control background.

Figure 5: The authors use bath application of Slit and Wnt to test effects of cables on Slit and Wnt responses. The observed effect sizes are very small and a single assay of this type does not allow such strong conclusions like 'loss of Cables prevents responsiveness.' Cables knockdown appears to lead to a strong reduction in axon growth (the effect is greater than the effect of Slit treatment). Could there be a ceiling/floor effect on the outgrowth response, such that the extent of outgrowth in cables ko can not be further enhanced by Slit addition? Perhaps growth cone collapse for Slit and growth cone turning for Wnt would give more convincing results.

We have rephrased the description of the assay, as it was obviously unclear and thus misunderstood by the reviewer. The results of our in vitro assays are in perfect agreement and explain the phenotypes that we see in vivo (lines 179ff). As shown previously (by different labs), the addition of Slit to post-crossing commissural axons results in collapse and re-growth attempts followed again by collapse, therefore, all- in-all shorter axons than in the absence of Slit. In contrast, addition of Wnt5a (Avilés and Stoeckli, Dev Neurobiol 76(2016)190-208) results in additional axon growth compared to controls. This is what we demonstrate with the green bars in Figure 5. The axons lacking dsCables1 do not reach the same average length as the control axons (dsCables1 compared to control). However, this is not a major issue of this figure. Rather, we demonstrate that axons lacking Cables1 cannot respond to Slit (that is, they are not shorter than the dsCables1 axons grown under control conditions, compare first two red bars).
Similarly, they also do not grow more in the presence of Wnt5a, as the dsCables1 axons with the addition of Wnt5a are the same length as those without Wnt5a added (compare 3rd red bar with the first red bar). The effect size is 21% shorter axons in the presence of Slit for control axons, but no effect for the dsCables1 axons (both dsCables1 and dsCables1&Slit are the same length). For the addition of Wnt5a, the control axons are 32% longer in the presence of Wnt5a. For the dsCables1 axons, the presence of Wnt5a does not change the average length (the average length in the presence of Wnt5a is 1% less than without Wnt5a present).

Figure 6: The authors should more clearly document the effects they are seeing in these manipulations. As written, all we know is that there are 'significant effects on axon guidance.' What are these effects? Do they see the predicted differences between robo/cables and B-catenin/cables phenotypes? e.g stalling defects in the case of robo and randomization of post-crossing turning in the case of B-catenin? (See also major comment above)

We wonder whether the reviewer looked at our revised version of the manuscript (submitted to Development) compared to the original version submitted to Reviewer Commons. In the version submitted to Development, we had added a qualitative analysis of the phenotypes (Figure 6C), as

© 2023. Published by The Company of Biologists under the terms of the Creative Commons Attribution License (https://creativecommons.org/licenses/by/4.0/).

suggested here again by the reviewer. We decided not to include images of the individual phenotypes again (as they are already documented in Figures 2 and 3 (as well as the movies).

The observed phenotypes are in agreement with our expectations that the combination of dsCables1 and dsRobo increased floorplate stalling, despite the fact that silencing Robo1 at E3 is not resulting in a very strong phenotype (see Alther et al., 2016 for explanation and details about the reduced efficiency of E3 versus E2 electroporation). Also, as expected, combined electroporation of dsCables1 and dsβCatenin resulted predominantly in no turns, in agreement with inhibition of Wnt signaling.

Also related to Figure 6:

The authors do not validate the dsRNA knockdown of either Robo or B catenin.

We have used the same dsRNAs in previous publications (dsRobo, see Alther et al., Development 143(2016)994-1004; dsβ-Catenin, see Avilés and Stoeckli, Dev Neurobiol 76(2016)190-208). Thus, we did not repeat the full validation. However, we did verify the efficiency of downregulation by the fact that we (NZ, AD, and GV compared to either T. Alther or E. Avilés) obtained the expected phenotypes with the same concentrations of dsRNA that we used previously (Supplementary Figure S5).

It is unclear what the interpretation or expectation of the triple knock down condition is.

We did not include the triple-knockdown condition. (Again, we wonder, whether the reviewer has really looked at the revised version of our manuscript, submitted to Development, rather than the original one submitted to Reviewer Commons). We would not expect any fundamental difference in phenotype: a mix between failing to cross the floorplate and no turns into the longitudinal axis. Therefore, we feel that this condition would not add anything to the conclusion of our studies.

Figure 7 and 8: The differences of phospho-catenin between proximal and distal in HH26 quantification suggests an ~30% increase. In contrast a nearly 4-fold increase is reported in Figure 8 for this same measurement. This raises concerns about the reproducibility of this pattern. Some comment on why this measurement is so different in the two ‘control’ conditions would be helpful. This is especially true given the very small effect of Robo Ko on P-Catenin levels shown in Figure 10

The experiments done in Figures 7 and 8 were not done at the same time and therefore the staining was not done with the same batch of anti- β-Catenin pY489. For this reason, the staining intensities were normalized and separate controls were used to be able to analyze the effect of Cables1. We have added an explanation in the Figure legend (Figure 8).

Also related to Figure 7 and 8:

Perhaps I missed it, but no validation of antibody specificity is provided or described.

The monoclonal antibodies against β-Catenin pY489 are the same that were used and specified in the original studies by Rhee et al. (Nature Cell Biol 9(2007)883-892), where the difference between different phosphorylation sites was carefully verified. The study was also done with chicken embryos. Thus, we do not think that the study by Rhee et al. needs to be repeated.

We have added some confirmatory staining in Supplementary Figure S7 demonstrating specificity of β- Catenin targeting. Knockdown of β-Catenin with a construct expressing an shRNA followed by IRES and EBFP demonstrated that effectively targeted Robo3-positive dI1 neurons showed much lower levels or no staining of either β-Catenin or β-Catenin pY489.

Figure 9: As for figure 5, phenotypic documentation is incomplete. In addition, no controls are shown to assure that the different mutant forms of B-catenin are comparably expressed, nor is there an unmutated wild-type control. It would be helpful to indicate on the histogram which comparisons lead to significant differences given the huge scatter of the data points.

© 2023. Published by The Company of Biologists under the terms of the Creative Commons Attribution License (https://creativecommons.org/licenses/by/4.0/).

Also here, we have to conclude that the reviewer did not look at our revised manuscript sent to Development but still comments on the original version that was submitted to Reviewer Commons. Figure 5 (Figure 6 in the revised manuscript) contains the detailed analysis of the different phenotypes that the reviewer asks for.

For Figure 9, we have added the analysis, as suggested. Furthermore, we have added Supplementary Figure S9 showing that both mutant forms of β-Catenin pY489 are expressed in dI1 neurons when expressed under control of Math1.

Finally, if as the authors suggest phosphorylation of B-catenin is what drives the switch from Slit response to Wnt response, why doesn't over-expression of the phosphomimetic B-catenin lead to aberrant premature anterior turning on the ipsilateral side of the spinal cord? Perhaps visualizing the distribution of the over-expressed B-cat variants as suggested above might offer some insight into this curiosity.

The Fzd3 receptor necessary for the response to Wnt is not expressed on growth cones of pre-crossing axons. As we demonstrated earlier (Alther et al., 2016), Fzd3 is inserted into the post-crossing growth cone membrane by specific trafficking in a Calsyntenin-1-dependent manner. Therefore, we would not expect any premature turning along the ipsilateral floor-plate border even in the presence of the phosphomimetic variant of β-Catenin.

Second decision letter

MS ID#: DEVELOP/2023/201671

MS TITLE: Cables1 links Slit/Robo and Wnt/Frizzled signaling in commissural axon guidance

AUTHORS: Esther Stoeckli, Nikole Zuniga, Alexandre Dumoulin, and Giuseppe Vaccaro
ARTICLE TYPE: Research Article

Thank you for sending your manuscript to Development through Review Commons.

I am happy to tell you that your manuscript has been accepted for publication in Development, pending our standard ethics checks.

Reviewer 1

Advance summary and potential significance to field

In this work, Authors provide new mechanistic insights into the modulation of guidance response during midline crossing.

Comments for the author

Authors responded to my main concerns.
Congratulations on this beautiful work.

Reviewer 2

Advance summary and potential significance to field

N/A

© 2023. Published by The Company of Biologists under the terms of the Creative Commons Attribution License (https://creativecommons.org/licenses/by/4.0/).

Comments for the author

N/A

Reviewer 3

Advance summary and potential significance to field

The authors identify a new player (Cables) in commissural axon guidance and provide evidence that cables may modulate both Slit and Wnt signaling in the developing spinal cord.

Comments for the author

Overall the authors have made substantial efforts to address my prior concerns. I still feel that some of the major conclusions with respect to Cables as a switch between Slit and Wnt signaling are overstated based on the experimental data (similar to reviewer 2). In addition, the explanation for the dramatically different effect sizes on phospho- B-catenin levels in different experiments is still not ideal- I can understand that signal levels may differ between batches of antibodies, but one would expect at least somewhat comparable fold-changes in signal. Perhaps the authors could tone down the phospho-switch argument a bit. Despite these reservations, the authors have done a nice job identifying and characterizing a new role for cables in commissural axon guidance.

© 2023. Published by The Company of Biologists under the terms of the Creative Commons Attribution License (https://creativecommons.org/licenses/by/4.0/).
